# Discovery of Potent Broad Spectrum Antivirals Derived from Marine Actinobacteria

**DOI:** 10.1371/journal.pone.0082318

**Published:** 2013-12-05

**Authors:** Avi Raveh, Phillip C. Delekta, Craig J. Dobry, Weiping Peng, Pamela J. Schultz, Pennelope K. Blakely, Andrew W. Tai, Teatulohi Matainaho, David N. Irani, David H. Sherman, David J. Miller

**Affiliations:** 1 Life Sciences Institute, University of Michigan, Ann Arbor, Michigan, United States of America; 2 Department of Internal Medicine, University of Michigan, Ann Arbor, Michigan, United States of America; 3 Department of Neurology, University of Michigan, Ann Arbor, Michigan, United States of America; 4 Department of Microbiology & Immunology, University of Michigan, Ann Arbor, Michigan, United States of America; 5 Department of Chemistry and Medicinal Chemistry, University of Michigan, Ann Arbor, Michigan, United States of America; 6 School of Medicine and Health Sciences, University of Papua New Guinea, Boroko, Papua New Guinea; Stazione Zoologica, Italy

## Abstract

Natural products provide a vast array of chemical structures to explore in the discovery of new medicines. Although secondary metabolites produced by microbes have been developed to treat a variety of diseases, including bacterial and fungal infections, to date there has been limited investigation of natural products with antiviral activity. In this report, we used a phenotypic cell-based replicon assay coupled with an iterative biochemical fractionation process to identify, purify, and characterize antiviral compounds produced by marine microbes. We isolated a compound from *Streptomyces kaviengensis*, a novel actinomycetes isolated from marine sediments obtained off the coast of New Ireland, Papua New Guinea, which we identified as antimycin A1a. This compound displays potent activity against western equine encephalitis virus in cultured cells with half-maximal inhibitory concentrations of less than 4 nM and a selectivity index of greater than 550. Our efforts also revealed that several antimycin A analogues display antiviral activity, and mechanism of action studies confirmed that these *Streptomyces*-derived secondary metabolites function by inhibiting the cellular mitochondrial electron transport chain, thereby suppressing de novo pyrimidine synthesis. Furthermore, we found that antimycin A functions as a broad spectrum agent with activity against a wide range of RNA viruses in cultured cells, including members of the *Togaviridae*, *Flaviviridae*, *Bunyaviridae*, *Picornaviridae*, and *Paramyxoviridae* families. Finally, we demonstrate that antimycin A reduces central nervous system viral titers, improves clinical disease severity, and enhances survival in mice given a lethal challenge with western equine encephalitis virus. Our results provide conclusive validation for using natural product resources derived from marine microbes as source material for antiviral drug discovery, and they indicate that host mitochondrial electron transport is a viable target for the continued development of broadly active antiviral compounds.

## Introduction

Infections caused by arthropod-borne viruses (arboviruses) represent dramatic examples of disease reemergence [1], due in part to significant urban growth as well as ease of worldwide travel, thereby producing conditions that facilitate arbovirus epidemics [2,3]. Furthermore, the threat posed by the intentional exposure of a population center to a virulent arbovirus has prompted the U.S. federal government to designate numerous arboviruses as high priority biodefense pathogens, particularly those that infect the central nervous system (CNS) causing encephalitis. The *Alphavirus* genus within the *Togaviridae* family contains about 30 mosquito-borne, enveloped, positive-stranded RNA viruses, one-third of which cause significant diseases in human and animals worldwide [4]. The encephalitic alphaviruses, including western, eastern, and Venezuelan equine encephalitis viruses (WEEV, EEEV, and VEEV), directly infect neurons resulting in CNS inflammation and neuronal destruction [5–8]. These highly virulent pathogens can cause severe disease in humans with fatality rates of up to 70%, as well as long-term neurological sequelae in most survivors [9,10].

There are currently no licensed vaccines or antiviral drugs for alphavirus infections. Formalin-inactivated vaccines for WEEV or EEEV and a live attenuated vaccine against VEEV (TC-83 strain) are available on an investigational drug basis, whose use is limited primarily to laboratory personnel working with these infectious agents. The development of alternative live attenuated, chimeric, and DNA-based alphavirus vaccines is being actively pursued, but the broad clinical application of these next generation vaccines is likely years away [11]. Furthermore, the combination of active vaccination plus antiviral therapy may be a more effective response in the setting of an outbreak due to either natural transmission or intentional exposure to a viral pathogen [12]. Although numerous compounds have been reported to inhibit alphavirus replication in cultured cells, only a select few have shown any activity in animal models [13–16]. Thus, there is a pressing need to identify new antiviral compounds and drug targets as part of an effective medical countermeasures strategy to prevent or mitigate illness, suffering, and death resulting from infections caused by these virulent pathogens [17].

Chemical libraries containing small molecule compounds with known structures provide a rich source of starting material for the identification of novel antiviral agents. Indeed, our group has used such libraries to identify a novel class of compounds effective against neurotropic alphaviruses [13,18]. Although these libraries can be vast in size and scope, their use is often constrained by factors such as the cost of acquiring or maintaining large compound collections and the limits of synthetic and combinatorial chemistry [19]. Even the largest small molecule libraries, often containing 10^6^ compounds or more, represent a vanishingly small fraction of the number of chemically feasible drug-like molecules, which is estimated to be on the order of 10^60^ to 10^100^ [20–22].

An alternative approach takes advantage of the complex biosynthetic pathways of living organisms, which can produce natural products of almost unlimited structural diversity [23]. This approach has been utilized quite effectively in the identification and development of antimicrobial agents, as a substantial portion of currently available drugs used clinically to treat bacterial and fungal infections were originally derived from microbial sources [24]. Although natural products have not previously been used to any large extent in the development of novel antiviral agents, there is limited precedence for this approach [14]. Numerous secondary metabolites obtained from microbes recovered over a range of geographical regions and habitats have been developed as potential therapeutics, and they are frequently the endpoint of a complex biosynthetic system that comprises a metabolic pathway [25]. These products have changed the face of human and veterinary medicine over the past several decades and continue to provide new drug leads for pharmaceutical development.

Sediment from shallow and deep water marine habitats is proving to be a rich source of novel microorganisms, particularly actinomycetes, whose metabolic products provide entirely new structural diversity with broad potential clinical applications [26–29]. For the current study, we used an established phenotypic cell-based replicon high throughput screen (HTS) and validation protocol [18] in conjunction with an extensive library of pre-fractionated extracts derived from marine actinomycetes [30] as starting material. This chemical diversity enabled us to implement a drug discovery program for antivirals effective against WEEV and related arboviruses. In this report, we describe an efficient bioassay-guided sequential fractionation process and purification of a natural product molecule generated by a novel marine *Streptomyces* species. We identify this compound as an antimycin A derivative, displaying potent and broad spectrum antiviral activity. We also provide evidence for its mechanism of action, which is mediated in part by disruption of mitochondrial electron transport and pyrimidine biosynthesis. Furthermore, we demonstrate that antimycin A reduces CNS viral titers and improves both clinical disease and survival in mice given a lethal challenge with WEEV. These results provide clear proof-of-concept that potent, broad spectrum antiviral compounds with in vivo activity against highly pathogenic arboviruses can be isolated and identified from natural product chemical diversity resources.

## Results

### Primary HTS and validation of candidate extracts from marine microbes

**Figure 1:**
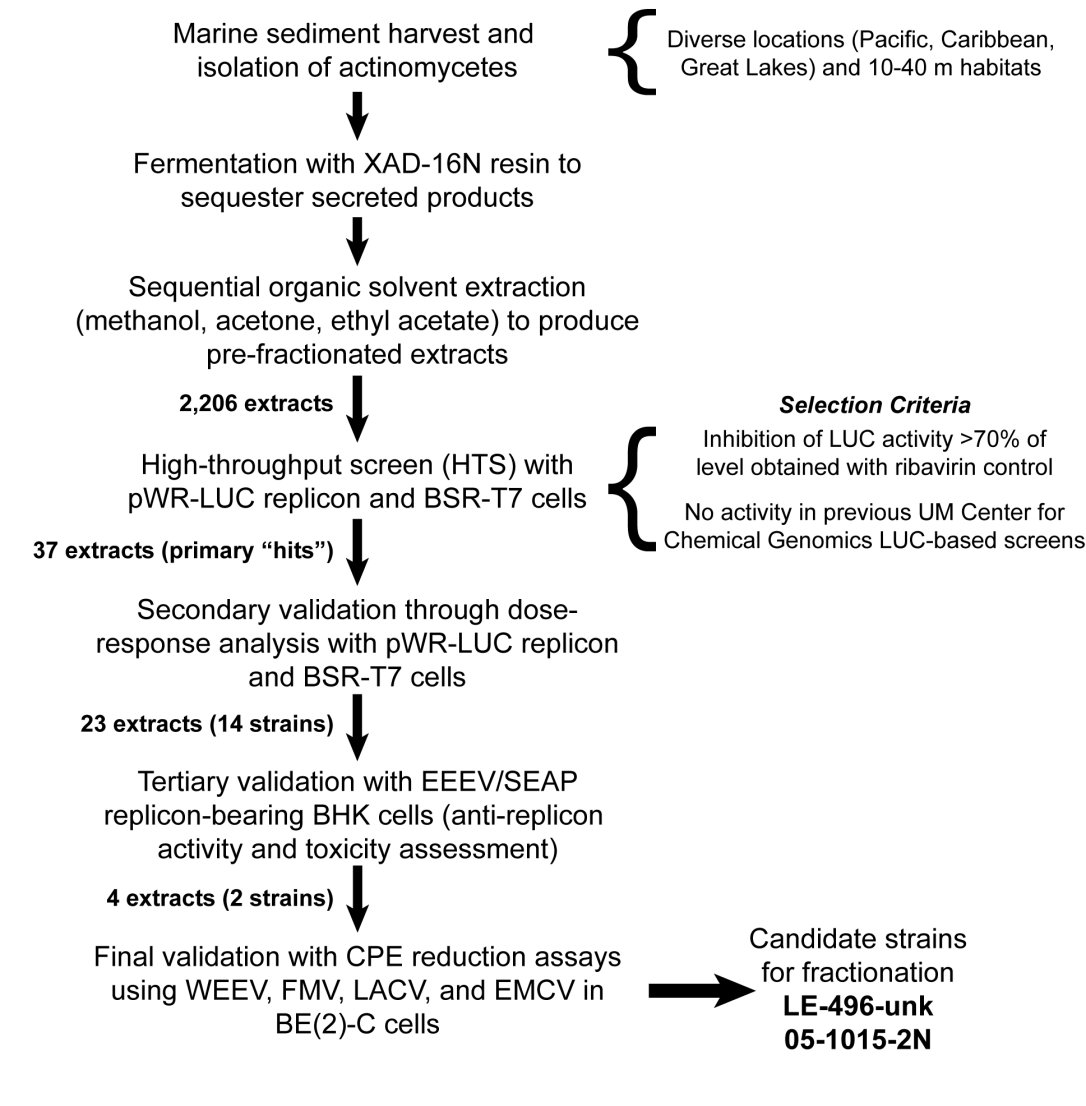
Schematic of marine microbe-based natural product extract production, screening, and validation. Individual steps are indicated in the left column, with explanatory comments provided on the right. The number of extracts and corresponding number of individual strains, where appropriate, are indicated in bold type between steps.

We previously developed, validated, and utilized a WEEV replicon cell-based assay to complete an HTS with a defined synthetic small molecule chemical library containing >50,000 compounds [18], leading to the development of a novel class of inhibitors with in vivo activity against neurotropic alphaviruses [13]. We used the same cell-based phenotypic assay to complete an additional HTS and secondary validation with a library of extracts derived from marine microbes isolated from diverse geographic regions. Figure 1 illustrates our workflow for strain purification and cultivation, extract preparation, HTS completion with selection criteria, and secondary, tertiary, and final validation steps. For each microbial isolate, which based on the location and isolation procedures are primarily within the order *Actinomycetales* [30], several pre-fractionated extracts were prepared by sequential organic solvent extraction of the resin used to adsorb the secreted metabolites.

We initially identified 37 extracts as primary hits from a collection of 2,206 extracts, and 23 of these extracts derived from 14 individual microbial isolates were validated in secondary dose-response assays using the WEEV replicon system. To further validate candidate extracts and select those with potential broad spectrum antiviral activity, we completed tertiary validation assays with an EEEV replicon bearing a secreted alkaline phosphatase reporter gene. We then used a final validation cytopathic effect (CPE) reduction assay with several infectious viruses, including the alphaviruses WEEV and Fort Morgan virus (FMV), the bunyavirus La Crosse virus (LACV), and the picornavirus encephalomyocarditis virus (EMCV). These rigorous validation steps led to the identification of four candidate extracts derived from two individual microbial isolates, designated LE-496-unk and 05-1015-2N. All remaining data contained in this report were generated with isolate 05-1015-2N, which we recovered from marine sediment collected near the southern coast of New Ireland province in Papua New Guinea. On the basis of 16S rRNA sequence, phylogenetic analyses, and geographic location where the isolate was recovered, we named this marine microbe *Streptomyces kaviengensis* (Figure S1).

### Isolation of a purified antiviral compound from the S. *kaviengensis*-derived extract

**Figure 2:**
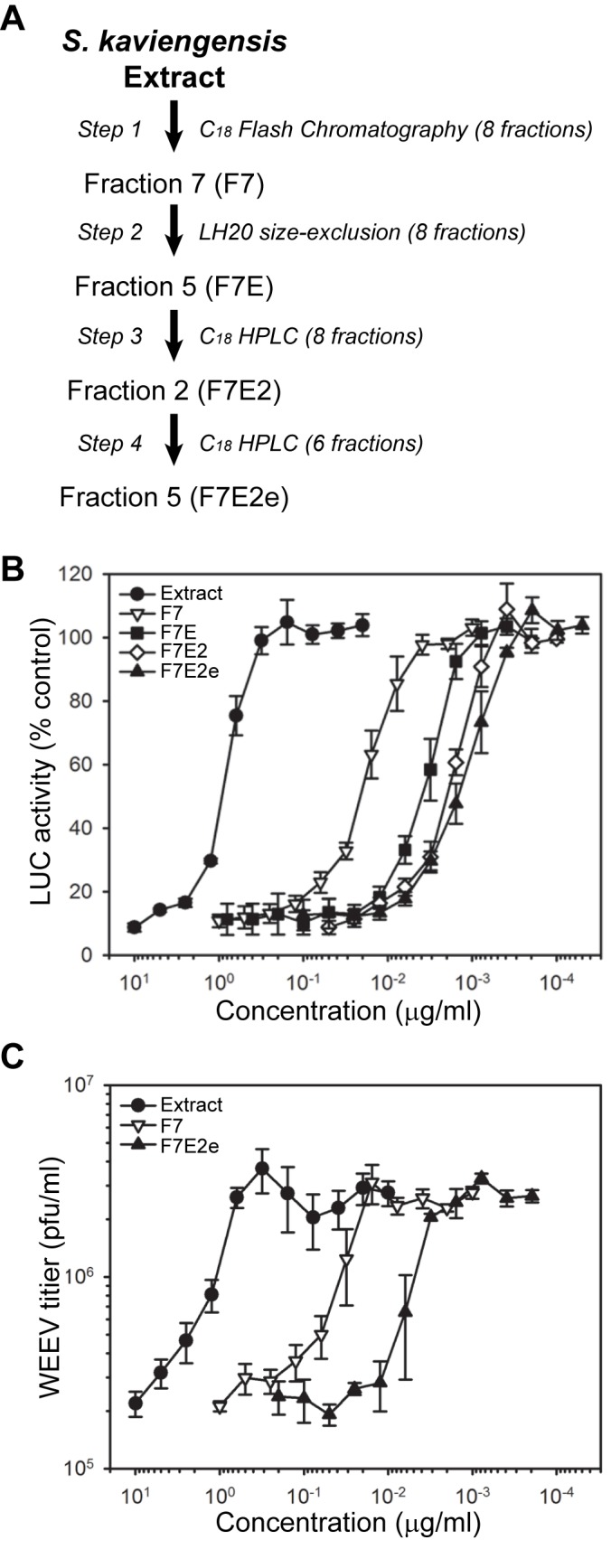
Sequential fractionation and purification of an active antiviral compound in the extract derived from *S. kaviengensis*. (A) Schematic of the four sequential fractionation steps used to obtain a purified antiviral compound from *S. kaviengensis*. Chromatographic method and total fractions from each separation step are shown in italics type. The individual fraction at each step that had the highest antiviral activity in the replicon assay was used for subsequent fractionation steps. The final purified compound is referred to as F7E2e. (B) Antiviral activity of the initial *S. kaviengensis* extract and sequential fractions analyzed with WEEV replicons. Results are presented as the percent untreated control transfected cells and represent the mean ± SEM from at least three analyses of fractions from one purification experiment. Similar results were obtained from a second independent purification experiment using the steps outlined in Figure 2A. (C) Antiviral activity of the initial *S. kaviengensis* extract and select sequential fractions analyzed with infectious WEEV in BE(2)-C neuronal cells. Cells were infected with WEEV at an MOI = 0.1, simultaneously treated with the indicated weight-based concentration of the indicated material (whole extract or specific fraction), and virus production was measured by plaque assay at 24 hpi. Results are presented as infectious virion concentration in tissue culture supernatants and represent the mean ± SEM from at least three analyses of fractions from one purification experiment. Similar results were obtained from a second independent purification experiment using the steps outlined in Figure 2A.

The S. *kaviengensis*-derived extract we identified and validated as possessing antiviral activity was most likely a complex mixture of secreted bacterial secondary metabolites containing an indeterminate number of distinct chemical entities. To isolate and purify the active compound or compounds responsible for the observed antiviral activity, we developed an empiric biochemical fractionation protocol that used the WEEV replicon assay to follow antiviral activity at each fractionation step (Figure 2). The first step involved C_18_ flash chromatographic separation of the S. *kaviengensis*-derived extract into eight fractions, which we initially used to confirm that inhibitory activity against WEEV replicons represented authentic antiviral activity against infectious virus (Figure S2). Fractions 6 and 7 contained the most potent inhibitory activity against WEEV replicons (Figure S2A), suppressed infectious WEEV production (Figure S2B), and rescued WEEV-induced CPE (Figure S2C), all indicating that the replicon assay was a valid and convenient surrogate to follow antiviral activity during subsequent fractionation steps.

We used four sequential separation steps for the final isolation of a purified antiviral compound from the S. *kanviengensis*-derived extract: C_18_ flash chromatography, LH-20 size exclusion, and two successive C_18_ HPLC steps (Figure 2A). We used weight-based concentrations and completed full dose-titration curves at each step to follow potency, thereby demonstrating a progressive increase in antiviral activity from initial extract to final pure active compound (designated F7E2e) in both the replicon inhibition assays (Figure 2B) and in the suppression of infectious WEEV titers (Figure 2C). To quantify sequential increases in activity and to examine potential toxicity, we calculated concentrations that produced a 50% reduction in cell viability (CC_50_), a 50% reduction in replicon activity or virus titers (IC_50_), or a 50% decrease in virus-induced CPE (EC_50_) (Table 1). This analysis revealed an approximate 500-fold increase in antiviral potency from the initial extract to the final active compound, F7E2e, and a selectivity index (CC_50_/IC_50_) for F7E2e of >550. Furthermore, there was excellent correspondence in the IC_50_ and EC_50_ values for individual samples, with nearly identical results for the final active compound F7E2e. Finally, HPLC and high resolution LC-MS analyses showed that F7E2e was >98% pure and had a molecular weight of 548.279 g/mol, which corresponded to IC_50_ and EC_50_ values of approximately 3 nM (Table 1).

**Table 1:** Quantitative antiviral potency values for *S. kaviengensis* extract and select fractions.

**Sample^*1*^**	**Toxicity (CC_50_)**	**Replicon LUC assay (IC_50_)**	**WEEV titers (IC_50_)**	**WEEV CPE reduction (EC_50_)**
Extract	>10,000^2^	900 ± 52	968 ± 11	1551 ± 95
Step 1 – F7	>10,000	19.7 ± 2.2	31.2 ± 7.5	9.5 ± 0.5
Step 4 – F7E2e	>1,000	1.8 ± 0.3	1.8 ± 0.2	1.7 ± 0.1
		(3.3 ± 0.5 nM)	(3.3 ± 0.3 nM)	(3.2 ± 0.3 nM)

^1^Correspond to samples in Figure 2A purification schematic.

^2^ Values in ng/ml represent the sample concentration that produced either a 50% reduction in replicon LUC activity or WEEV titers or a 50% increase in virus-infected cell viability at 24 hpi compared to untreated controls. For toxicity values, the highest concentration used in the titration assays is shown. Results are presented as the mean ± SEM from at least three independent experiments. Molar IC_50_ and EC_50_ values for F7E2e are given in parentheses and are based on a MW of 548.279 g/mol determined by LC-MS.

**Figure 3:**
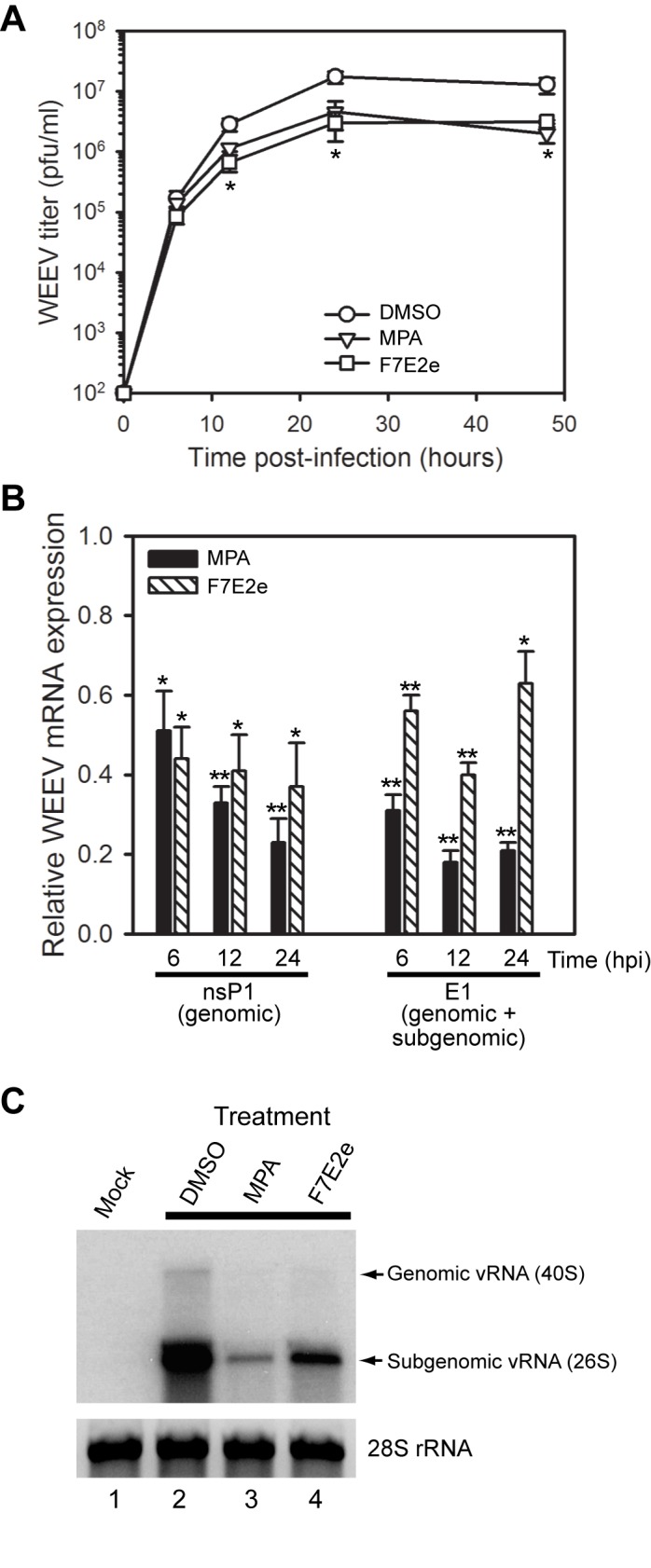
Purified antiviral compound from *S. kaviengensis* suppresses WEEV RNA replication and virus production in single-step growth assays. (A) Infectious virion production. BE(2)-C cells were infected with WEEV at an MOI = 10, treated with DMSO, 25 μM mycophenolic acid (MPA), or 100 ng/ml (~200 nM) purified compound F7E2e, and virus titers is tissue culture supernatants were determined by plaque assay at 6, 12, 24, and 48 hpi. Plaque assay sensitivity was 10^2^ pfu/ml. Results represent the mean ± SEM from three independent experiments. *p-value < 0.05 compared to DMSO-treated controls for both MPA- and F7E2e-treated samples. (B) Quantitative RT-PCR analysis of WEEV RNA accumulation. Cells were infected and treated as above in (A), total RNA was harvested at the indicated time points, and primers corresponding to either the nsP1 or E1 WEEV genome were used to amplify and quantify either genomic (nsP1) or genomic plus subgenomic (E1) RNA accumulation. Results are presented as WEEV RNA levels relative to infected DMSO-treated control cells, and represent the mean ± SEM from six independent experiments. *p*-value < 0.001* or 0.0001** compared to DMSO-treated controls. (C) Northern blot analysis of WEEV RNA accumulation. Mock-infected cells (lane 1) or cells infected and treated as above in (A) with DMSO (lane 2), MPA (lane 3) or F7E2e (lane 4) were harvested at 12 hpi, and total RNA was analyzed by Northern blotting with a strand-specific ^32^P-labelled riboprobe that detected both positive-sense genomic and subgenomic viral RNA (vRNA). The location and relative size of genomic and subgenomic vRNA are shown on the right, and the ethidium bromide-stained 28S rRNA band is shown as a loading control. Representative results from one of three independent experiments are shown.

We confirmed the antiviral activity of F7E2e against WEEV in single-step growth assays using BE(2)-C human neuronal cells infected with virus at a multiplicity of infection (MOI) = 10 (Figure 3). For these experiments we used F7E2e at 100 ng/ml (~200 nM) and included mycophenolic acid as a positive control, as this cellular inosine 5’-monophosphate dehydrogenase inhibitor effectively suppresses alphavirus replication [31]. Both mycophenolic acid and F7E2e reduced infectious WEEV production by 12 h post-infection (hpi) and resulted in a stable 10-fold reduction in virus titers at 24-48 hpi (Figure 3A). We also measured viral RNA accumulation by qRT-PCR (Figure 3B) and northern blotting (Figure 3C), and found that F7E2e suppressed the accumulation of both viral genomic and subgenomic RNA in infected cells.

**Figure 4:**
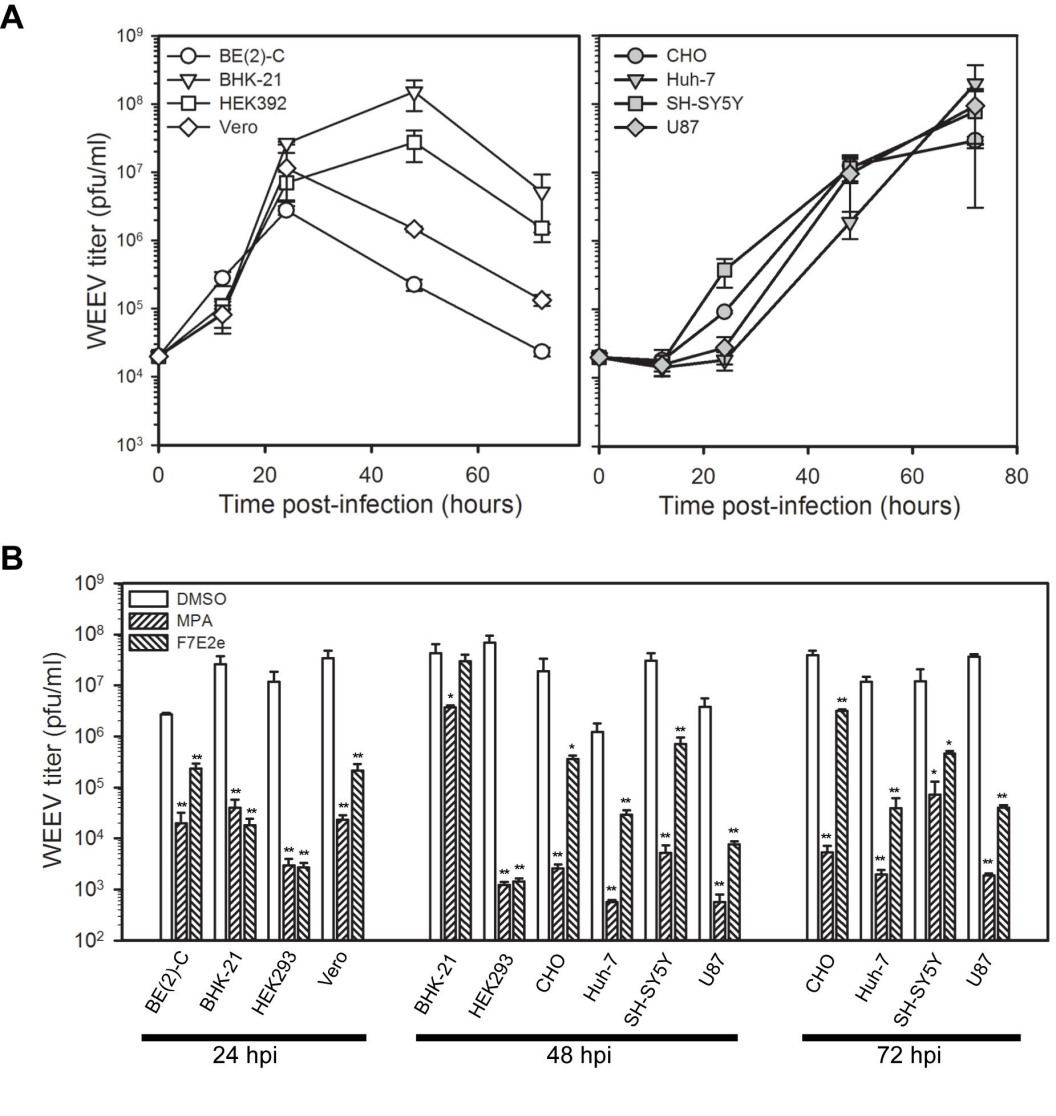
Purified antiviral compound from *S. kaviengensis* has potent but variable antiviral activity against WEEV in a wide range of cell lines. (A) Time course of WEEV production in various mammalian cell lines. Individual cell lines were infected with WEEV at an MOI = 0.1 and infectious virus titers in tissue culture supernatants were determined at 24, 48, and 72 hpi by plaque assay. WEEV-infected MDBK, HeLa, and A549 cells were also examined but showed no significant virion production by 72 hpi (data not shown). Results represent the mean ± SEM from three independent experiments. (B) Antimycin A activity against WEEV in various mammalian cell lines. The indicated cell lines were infected as described above, simultaneously treated with DMSO control, 25 μM mycophenolic acid (MPA), or 100 ng/ml (~200 nM) F7E2e, and infectious virus titers in tissue culture supernatants were determined at the indicated time post-infection. The drug concentrations used for treatment were 50- to 100-fold the IC_50_ values for each compound in the WEEV replicon assay (see Table 1 and Figure 5). Results represent the mean ± SEM from three independent experiments. *p*-value < 0.05* or 0.005** compared to DMSO-treated controls.

To further characterize the antiviral activity of F7E2e, we examined its activity against WEEV in several cell lines derived from various mammalian species and tissues (Figure 4 and Table 2). We infected cells at a low inoculum (MOI = 0.1) and determined the optimal time point to harvest supernatants for WEEV titer analysis. The temporal pattern of WEEV production varied between cell lines, with maximal production of infectious virions at 24 hpi for BE(2)-C and Vero cells, 24 to 48 hpi for BHK-21 and HEK293 cells, and 48 to 72 hpi for CHO, Huh-7, SH-SY5Y, and U87 cells (Figure 4A). We subsequently infected cells with WEEV, simultaneously treated with either mycophenolic acid or F7E2e, and harvested supernatants at the appropriate times post-infection for individual cell lines to analyze virus titers (Figure 4B). While both compounds suppressed infectious WEEV production in all cell lines tested, the magnitude of suppression varied between cell lines from approximately 200- to 9,000-fold for mycophenolic acid and 15- to 5,000-fold for F7E2e (Table 2). This variance was not due to differential compound toxicity (Figure S3) or intrinsic characteristics of the cell lines, such as the expression of drug efflux pumps, as the pattern of suppression varied between compounds. For example, although both compounds potently suppressed WEEV production in HEK293 cells, their activity in CHO, Huh-7, and SH-SY5Y cells was quite divergent (Table 2). Taken together, these results indicated that we had successfully isolated and purified a compound produced by *S. kaviengensis* with potent antiviral activity against WEEV.

**Table 2:** WEEV titer reduction mediated by *S. kaviengensis*-derived compound F7E2e is cell line-dependent.

			**WEEV titer fold-reduction^*1*^**	
**Cell line**	**Species**	**Tissue/cell type**	**Mycophenolic acid**	**F7E2e**
BE(2)-C	Human	Neuron	179	14
BHK-21	Hamster	Kidney fibroblast	920	1,391
HEK293	Human	Kidney epithelium	5,107	4,968
Vero	Primate	Kidney epithelium	1,419	166
CHO	Hamster	Ovarian epithelium	5,665	42
Huh-7	Human	Hepatocyte	2,030	57
SH-SY5Y	Human	Neuron	8,568	59
U87	Human	Astrocyte	8,156	463

^1^ Values represent the average decrease in infectious WEEV production in cells treated with 25 μM mycophenolic acid or 100 ng/ml (~200 nM) *S. kaviengensis*-derived compound F7E2e. Tissue culture supernatants were harvested at 24 hpi for BE(2)-C, BHK-21, HEK293, and Vero cells, and 48 hpi for CHO, Huh-7, SH-SY5Y, and U87 cells (see Figure 4).

### Antimycin A derivatives produced by *Streptomyces* species are potent antivirals against WEEV serogroup alphaviruses

**Figure 5:**
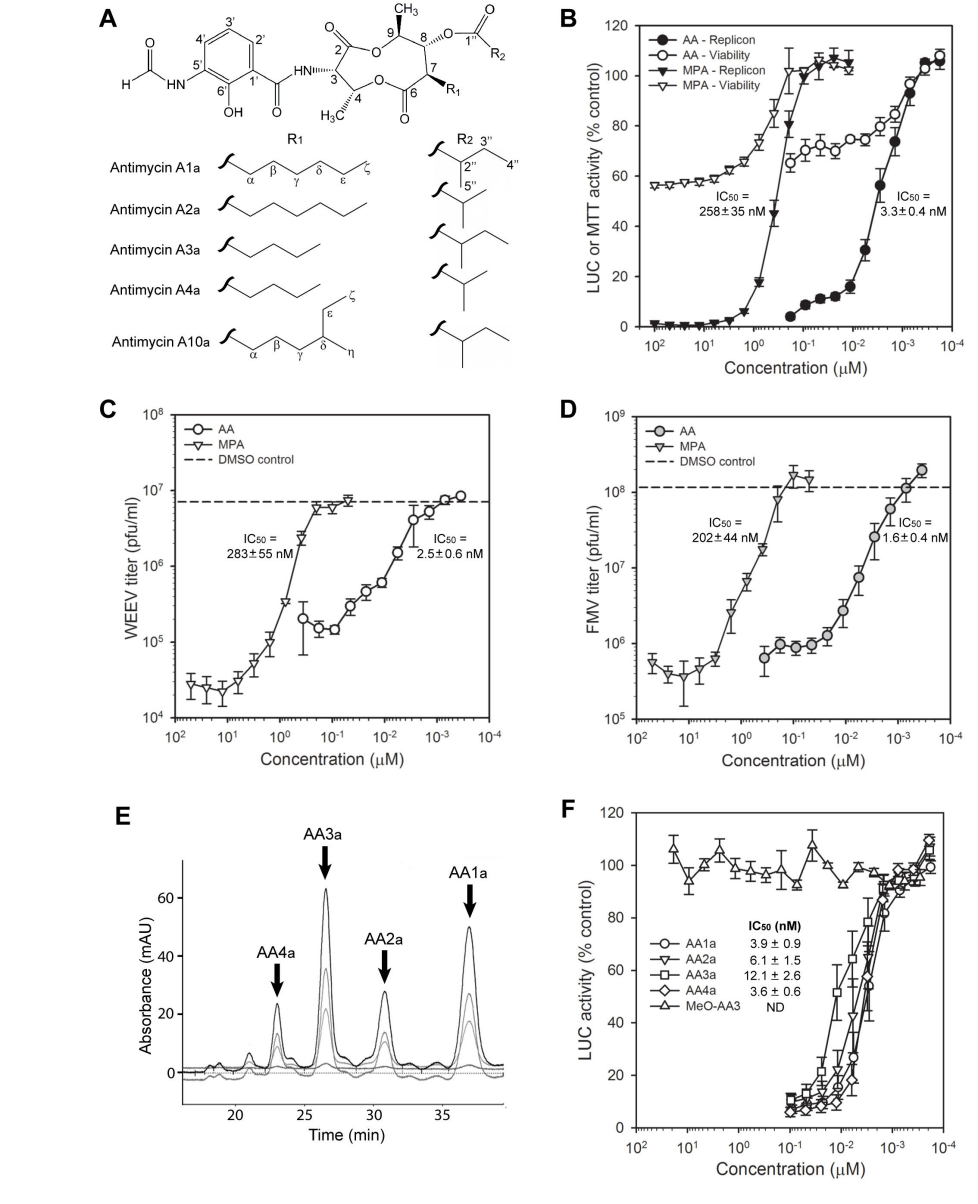
Antimycin A derivatives produced by *Streptomyces* have potent antiviral activity against WEEV serogroup alphaviruses. (A) Molecular structure of antimycin A. Core structure is shown at the top, and the individual R1 and R2 constituents of derivatives A1a, A2a, A3a, A4a, and A10a are shown below the core structure. Specific atom designations correspond to the NMR results in Table S1. (B) Antiviral activity of commercial antimycin A (AA) and mycophenolic acid (MPA) analyzed with WEEV replicons. Dose titration results for both replicon activity (closed symbols) and viability (open symbols) are presented as the percent untreated control cells and represent the mean ± SEM from at least five independent experiments. Calculated IC_50_ values for anti-replicon activity are shown on the graph for both compounds, and an average MW of 550 g/mol was used to estimate molar concentrations for commercial antimycin A. (C and D) Antiviral activity of commercial AA and MPA analyzed with infectious WEEV (C) or FMV (D) in BE(2)-C neuronal cells. Cells were infected with WEEV (MOI = 0.1) or FMV (MOI = 1), treated simultaneously with compounds at the indicated concentrations, and virus production was measured by plaque assay at 24 hpi. Results are presented as infectious virion concentration in tissue culture supernatants and represent the mean ± SEM from at least three independent experiments. Calculated IC_50_ values are shown on the graph for both compounds, and for commercial antimycin A these values were determined as described above in (B). The dashed reference lines represent results from infected cells treated with DMSO control. (E) HPLC separation of individual antimycin A derivatives from commercial stock compound. Only the select portion of an HPLC tracing that contained the four most prominent peaks is shown, and the various grey scale tracings represent different absorbance wavelengths. The identification of individual antimycin A derivatives represented by the four most prominent peaks is shown, where structures were determined by NMR analysis of purified fractions (see Table S1). (F) Antiviral activity of individual antimycin A derivatives analyzed with WEEV replicons. Dose titration results are presented as the percent untreated control cells and represent the mean ± SEM from at least four independent experiments. Calculated IC_50_ values for individual derivatives are shown on the graph, and were calculated using MWs of 548.63, 534.61, 520.58, and 506.55 g/mol for antimycins A1a, A2a, A3a, and A4a, respectively. The methoxy group in 2-methoxyantimycin A3 (MeO-AA3) is located at the 6’ position in the core antimycin structure shown in Figure 5A. ND, not determined.

We confirmed the molecular structure of F7E2e by an extensive array of 1D and 2D NMR techniques and high resolution MS analysis, ultimately determining that the antiviral compound was antimycin A1a (Figure 5A and Table S1). We determined the structure of a second purified compound from *S. kaviengensis* (F7E2f) as antimycin A10a, which also displayed potent antiviral activity against WEEV replicons with an IC_50_ of approximately 3 nM (data not shown). Antimycins are a family of secondary metabolites produced by *Streptomyces* species, consisting of a 3-formylaminosalicylic acid linked via an amide bond to a nine-membered cyclic dilactone moiety, plus two alkyl side chains that vary in length and composition (Figure 5A). These natural products were first isolated in 1949 [32], and have been shown to possess antifungal [33–35] and some antiviral [36–38] activity. The antimycins block the mitochondrial electron transport chain (mETC) and hence cellular respiration by binding the Q_i_ site of cytochrome C reductase in complex III [39]. Thus, antimycins can have profound effects on cellular physiology, including significant cytotoxicity at moderate to high doses. Indeed, antimycin A is the active ingredient of Fintrol, a piscicide commonly used in fishery management [40].

To verify that antimycin A functions as an antiviral against WEEV, we examined the activity of an authentic standard (Sigma-Aldrich A8674) in the replicon assay (Figure 5B). Commercial antimycin A showed potent inhibitory activity against WEEV replicons, with an IC_50_ of approximately 3 nM, which was almost 100-fold more potent than mycophenolic acid (Figure 5B, closed symbols). Although antimycin A showed cellular toxicity similar to mycophenolic acid, cell viability measured by 3-[4,5-dimethylthizol-2-yl]-2,5-diphenyltetrazolium bromide (MTT) signal was never less than 50% of control even at the highest concentrations tested (Figure 5B, open symbols). We obtained similar results using trypan blue exclusion and cell counting to measure cytotoxicity (data not shown). We further investigated the antiviral activity of antimycin A in BE(2)-C cells infected with WEEV (Figure 5C) or FMV (Figure 5D), a lower virulence WEEV serogroup alphavirus that can be safely handled under BSL2 conditions [41]. Antimycin A reduced WEEV and FMV titers by 10- to 100-fold, with IC_50_ values similar to those obtained in the replicon assay (Figure 5B) and with compound F7E2e (Table 1). Antimycin A was also approximately 100-fold more potent than mycophenolic acid in suppressing WEEV and FMV titers, similar to the replicon assay results, although the maximal level of suppression for WEEV titers was greater with mycophenolic acid (Figure 5C).

The commercial antimycin A standard is a mixture of congeners isolate from *Streptomyces* species with four major components (manufacturer’s product literature). We verified this by HPLC (Figure 5E), purified the four major peaks, and confirmed their structures by NMR (Table S1). We identified the four major components as antimycins A1a, A2a, A3a, and A4a (Figures 5A and 5E), all of which showed potent individual activity against WEEV replicons (Figure 5F). We also examined the antiviral activity of a commercial analogue, 2-methoxyantimycin A3, which has been developed as a potential anticancer agent due to potent inhibition of the anti-apoptotic protein, Bcl-x_L_ [42,43]. Modification of the 3-formylaminosalicyclic acid moiety disrupts antimycin A binding to cytochrome C reductase in complex III and abrogates inhibition of the mETC and cellular respiration, thereby reducing its toxicity [44,45]. However, 2-methoxyantimycin A3 was completely ineffective in suppressing WEEV replicon activity (Figure 5F), suggesting that mETC inhibition played a central role in the antiviral activity of antimycin A.

### Antimycin A1a purified from *S. kaviengensis* and commercial antimycin A standard induce similar transcriptional responses indicative of mitochondrial dysfunction

The previous identification of the mETC complex III as the target for antimycin A [39], and the WEEV replicon results with the 2-methoxyantimycin A3 derivative (Figure 5F), provided strong evidence for its potential mechanism of action. However, mETC inhibition likely has pleotropic effects within cells, and the nanomolar IC_50_ values we identified for antimycin A (Figure 5 and Table 1) were substantially lower than the concentrations often used for cell-based assays [36,38,42,46]. Thus, to assess the cellular response to antimycin A treatment we conducted genome-wide transcriptional microarray analyses. This approach provided further evidence supporting the identification of F7E2e derived from *S. kaviengensis* by comparative transcriptional response analyses with commercial antimycin A. We identified 1,120 up-regulated and 35 down-regulated genes in BE(2)-C cell treated with F7E2e (Table S2), and 976 up-regulated and 45 down-regulated genes in the same cells treated under identical conditions with commercial antimycin A (Table S3). There was a 61% concordance in the gene sets between the two treatment groups when all genes regulated ≥2-fold were compared, which increased to 73% concordance when only genes regulated ≥3-fold were analyzed (Table S4). There were 757 genes co-regulated in BE(2)-C cells treated with F7E2e or commercial antimycin A, and there was a significant correlation (R = 0.96) in the magnitude of transcriptional changes of individual genes in cells treated with either of the two compounds (Table S4 and Figure S4).

To further analyze the cellular changes induced in BE(2)-C cells by F7E2e or commercial antimycin A, we conducted in silico analyses with differentially regulated genes that were assigned to known cellular pathways using Ingenuity Pathway Analysis software. We identified 46 canonical pathways preferentially modulated after treatment with F7E2e and 45 preferentially modulated after treatment with commercial antimycin A, with 32 pathways overlapping between the two treatments (Table S5). Table 3 lists the ten canonical pathways co-modulated by both F7E2e and commercial antimycin A that showed the highest significance. The most significantly associated pathway, by an overwhelming margin, was the mitochondrial dysfunction pathway, which included numerous mETC components co-regulated after treatment with F7E2e or commercial antimycin A (Table S6). The strong correlation in cellular responses between treatments supported the molecular identification of F7E2e isolated from *S. kaviengensis* as antimycin A1a. Furthermore, although we cannot exclude the modulation of non-mitochondrial pathways as potential mechanisms, these results suggested that mETC disruption was a primary mechanism behind the antiviral activity of antimycin A.

**Table 3:** Cellular pathways modulated in BE(2)-C cells treated with *S. kaviengensis*-derived compound F7E2e or commercial antimycin A.

		***p*-value from IPA analysis**	
**Ingenuity Canonical Pathway^*1*^**	**Molecular/Cellular Functions**	***S. kaviengensis* F7E2e**	**Antimycin A**
Mitochondrial dysfunction	Free radical scavenging; Cellular function and maintenance	2.0 x 10^-20^	1.3 x 10^-13^
Colanic acid building block synthesis	Energy production	0.00056	0.0033
2-ketoglutarate dehydrogenase complex	Energy production; Lipid metabolism	0.00058	0.00040
TCA cycle II (eukaryote)	Free radical scavenging; Small molecule biochemistry; Lipid metabolism	0.0010	0.000006
2-oxobutanoate degradation I	Small molecule biochemistry; Lipid metabolism; Vitamin/mineral metabolism	0.0014	0.00095
Arginine biosynthesis IV	Cellular growth and proliferation; Amino acid metabolism	0.0026	0.0018
Superpathway of cholesterol biosynthesis	Small molecular biochemistry; Lipid metabolism	0.0037	0.0020
Role of BRCA1 in DNA damage response	DNA replication, recombination, and repair; Cell death and survival	0.0042	0.0066
Ascorbate recycling (cytosolic)	Energy production; Lipid metabolism	0.0081	0.0063
Mismatch repair in eukaryotes	DNA replication, recombination, and repair	0.0087	0.0055

^1^ The listed pathways were identified as significantly associated with transcriptional profile changes induced in BE(2)-C cells treated for 24 h with 100 ng/ml (~200 nM) F7E2e or commercial antimycin A purchased from Sigma-Aldrich, using Ingenuity Pathway Analysis (IPA) software. Only those pathways identified with *p*-values < 0.01 for both treatments are shown. A complete list of all IPA pathways associated with treatment using either compound at a threshold *p*-value of < 0.05 is provided in Table S5.

Sublethal and selective disruption of mETC function inhibits virus replication in part due to suppression of de novo pyrimidine synthesis.

**Figure 6:**
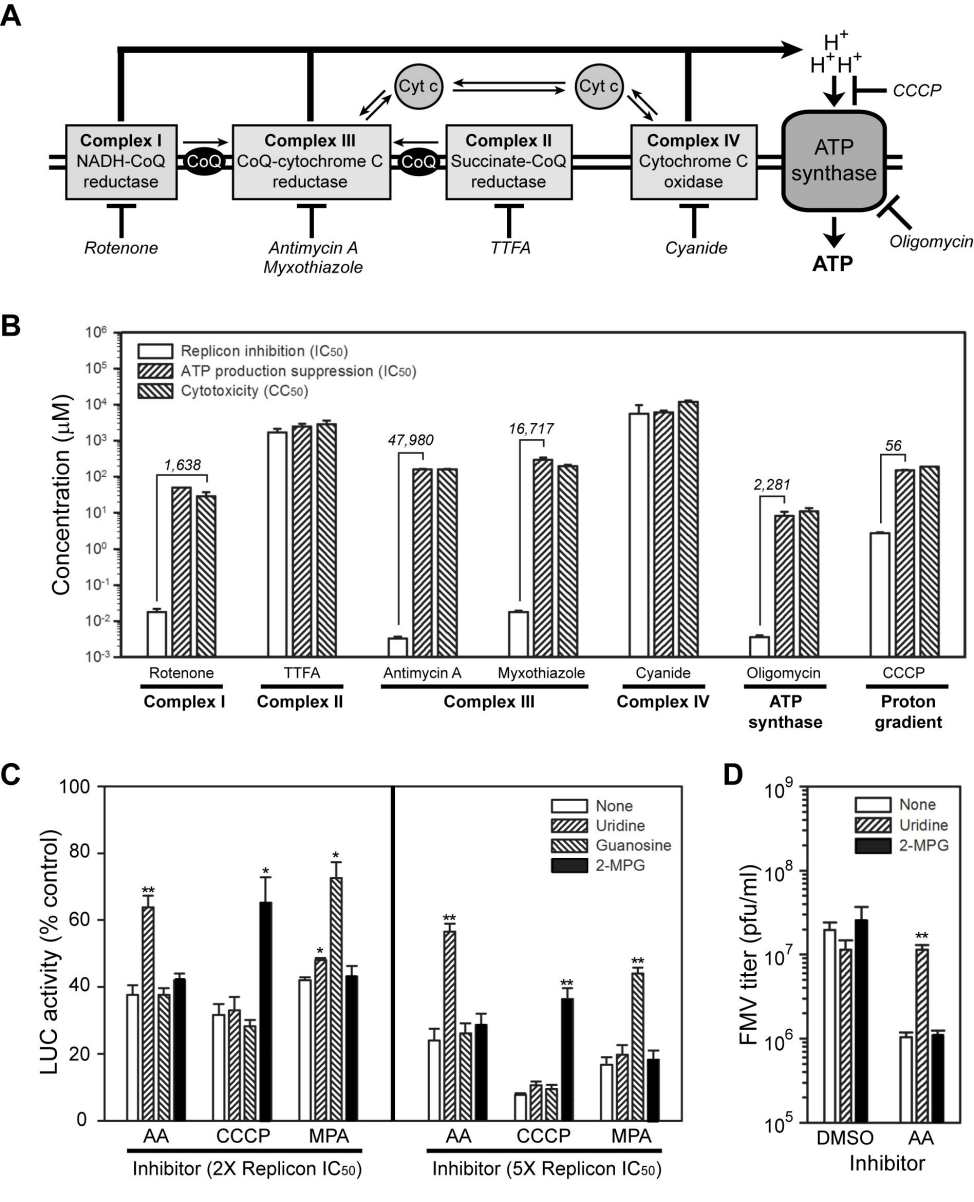
Disruption of mitochondrial electron transport suppresses WEEV replication. (A) Schematic of mETC enzyme complexes. The known targets for the inhibitors shown in italics are indicated by the cross bars. Cyt c, cytochrome C; CoQ, coenzyme Q. (B) Antiviral activity and toxicity of mETC inhibitors. Cells were treated with increasing concentrations of the indicated inhibitors, and replicon inhibition, total cellular ATP production, and cytotoxicity were measured in separate assays. Results are presented as IC_50_ or CC_50_ values for the indicated parameter, and represent the mean ± SEM from at least three independent experiments. The numerical values on the graph indicate fold-differences in IC_50_ values between replicon inhibition and ATP production suppression for the indicated select compounds. For rotenone, the comparison was made with CC_50_ values, since we were unable to calculate reliable IC_50_ values for ATP production suppression. (C) Complementation assays with select mETC inhibitors and WEEV replicons. Cells were treated with 100 μM of the indicated supplement or antioxidant and antimycin A (AA), CCCP, or mycophenolic acid (MPA) at 2X or 5X replicon IC_50_ concentrations, and replicon activity was measured 16-20 h later. Results represent the mean ± SEM from four independent experiments. *p*-value < 0.05* or 0.005** compared to supplement- or antioxidant-only treated controls. 2-MPG, *N*-(2-mercaptopropionyl)glycine. (D) Complementation assay with antimycin A and infectious virus. BE(2)-C cells were infected with FMV at an MOI = 1, treated simultaneously with 100 μM of the indicated supplement or antioxidant and control DMSO or antimycin A at 5X replicon IC_50_ concentration, and viral titers in tissue culture supernatants were measured at 24 hpi. Results represent the mean ± SEM from four independent experiments. **p-value < 0.005 compared to inhibitor-treated controls without supplementation (open bars).

We focused subsequent mechanism of action studies designed to understand the antiviral activity of antimycin A on the mETC (Figure 6). Figure 6A shows a schematic of the mETC, which resides in the mitochondrial inner membrane and is responsible for oxidative phosphorylation, respiration, and aerobic ATP production in eukaryotes. There are three enzyme complexes responsible for electron transport and electrochemical proton gradient maintenance: NADH-coenzyme Q (CoQ) reductase (complex I), CoQ-cytochrome C reductase (complex III), and cytochrome C oxidase (complex IV). Two additional enzyme complexes also participate in oxidative phosphorylation: succinate-CoQ reductase (complex II), which links the mETC to the citric acid cycle via reduction of CoQ, and ATP synthase, also referred to as complex V, which is ultimately responsible for protein gradient-driven ATP production. There are known inhibitors for all five enzyme complexes, which are shown at their site of action in Figure 6A. Furthermore, there are two well-described complex III inhibitors: antimycin A and myxothiazole, which block the Q_i_ and Q_o_ sites of cytochrome C reductase, respectively [39]. The proton ionophore carbonyl cyanide 3-chlorophenylhydrazone (CCCP) also disrupts mETC activity via bypassing proton efflux through the ATP synthase complex.

We used the seven inhibitors shown in Figure 6A to examine the impact of suppression at different sites within the mETC on WEEV replicon activity (Figure 6B). The complex I inhibitor, rotenone, the complex III inhibitors, antimycin A and myxothiazole, and the ATP synthase inhibitor, oligomycin, all potently inhibited WEEV replicon activity with IC_50_ values in the low nanomolar range (Figure 6B, open bars). The proton ionophore, CCCP, was less active and inhibited replicon activity with an IC_50_ of approximately 3 μM, whereas replicon inhibition IC_50_ values for the complex II inhibitor, thenoyltrifluoroacetone, and the complex IV inhibitor, cyanide, were in the low millimolar range. Eukaryotic mETC activity is responsible for ATP production under aerobic conditions, and therefore the antiviral activity of inhibitors may result from a decrease in energy production and non-selective viral suppression. To examine this potential confounding effect of mETC inhibition, we determined IC_50_ values for global ATP suppression and CC_50_ values for toxicity (Figure 6B, cross-hatched bars), and calculated ATP IC_50_/replicon IC_50_ ratios for each inhibitor. For thenoyltrifluoroacetone and cyanide, there were no significant differences in IC_50_ or CC_50_ values for replicon inhibition, ATP production suppression, or cytotoxicity. In contrast, CCCP had a ratio of 56, rotenone and oligomycin had ratios of 1,638 and 2,281, respectively, and myxothiazole and antimycin A had the largest ratios of 16,717 and 47,980, respectively. These results indicated that some, but not all, mETC inhibitors disrupted WEEV replicon activity in the absence of global ATP suppression or overt cellular toxicity, and that complex III inhibitors had the most potent and selective activities.

We further examined the effect of mETC suppression of WEEV replicon activity using combination treatments to examine possible synergy or antagonism between inhibitors. We used WEEV replicons and pairwise combination treatments with antimycin A and rotenone, myxothiazole, oligomycin, or CCCP to calculate Chou-Talaley parameters and combination index values [47]. We found no synergy with any combination treatment, near additive effects with antimycin A and rotenone, myxothiazole, or oligomycin, but strong antagonism between antimycin A and CCCP (Table S7). Complex I and III in the mETC are both significant sources of reactive-oxygen species (ROS) [48,49], whereas mitochondrial uncoupling by proton gradient disruption can either prevent or enhance mitochondrial ROS production in a dose-dependent and tissue- or cell type-specific manner [49–51]. These observations suggested that antimycin A-CCCP antagonism was due to differential effects on ROS generation. We were unable to detect significant ROS generation using either compound at concentrations up to 5X the IC_50_ for replicon inhibition, whereas 1,000-fold higher antimycin A concentrations readily induced detectable ROS (data not shown), as previously reported [46].

We subsequently used complementation assays and the glutathione analogue antioxidant *N*-(2-mercaptopropionyl)glycine (2-MPG) to examine the possible functional significance of low level ROS generation on antimycin A- or CCCP-mediated inhibition of WEEV replicon activity (Figure 6C). We also used uridine and guanosine for complementation experiments, as cellular pyrimidine and purine biosynthesis pathways are potential antiviral drug targets [52–54], and de novo pyrimidine biosynthesis is tightly linked to the mETC via dihydroorotate dehydrogenase (DHODH) [55]. Uridine, guanosine, and 2-MPG had no impact on cell viability or replicon activity in the absence of mETC inhibitors (data not shown). As expected, guanosine rescued mycophenolic acid-induced WEEV replicon suppression [31,54], whereas neither uridine nor 2-MPG produced significant complementation. For antimycin A-treated cells, only uridine rescued WEEV replicon activity, whereas only 2-MPG rescued activity in CCCP-treated cells. We obtained similar results using another antioxidant, *N*-acetyl cysteine (data not shown). Finally, we confirmed the replicon complementation results using infectious virus, where we found that uridine supplementation rescued antimycin A-mediated suppression of FMV production in BE(2)-C cells (Figure 6D). These results suggested that antimycin A and CCCP inhibited WEEV replicon activity via distinct mechanisms, and that suppression of de novo pyrimidine synthesis was partially responsible for the antiviral activity of antimycin A.

### Antimycin A has broad spectrum antiviral activity against a range of RNA viruses

**Figure 7:**
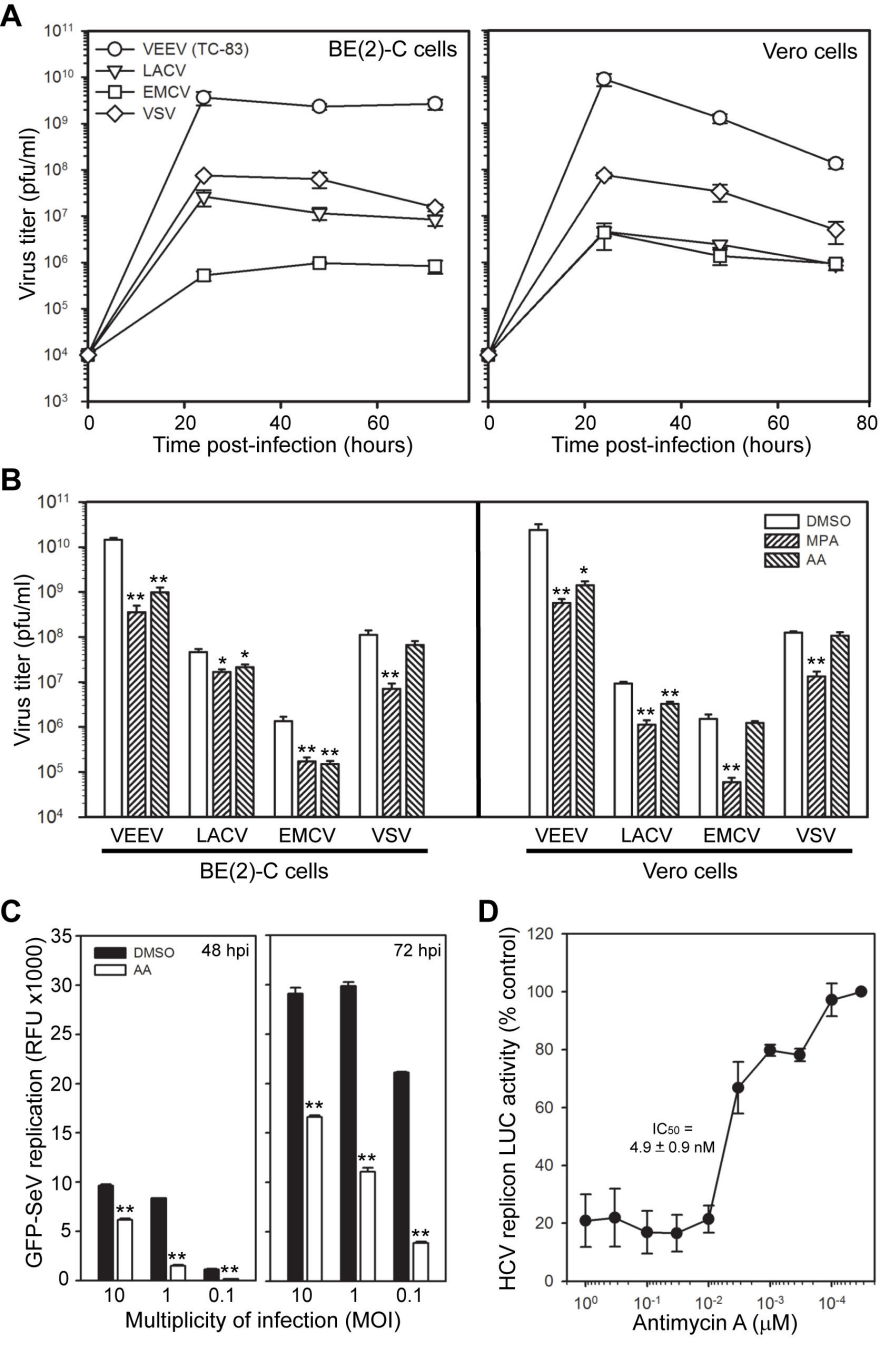
Antimycin A has broad spectrum antiviral activity against RNA viruses. (A) Time course of virion production. BE(2)-C (left graph) or Vero (right graph) cells were infected with the indicated viruses at an MOI = 0.1 and virus titers in tissue culture supernatants were determined by plaque assay at 24, 48, and 72 hpi. The input virus concentration was 10^4^ pfu/ml. (B) Antimycin A activity against VEEV, LACV, EMCV, and VSV. BE(2)-C (left graph) or Vero (right graph) cells were infected with the indicated viruses at an MOI = 0.1, treated simultaneously with DMSO, 25 μM mycophenolic acid (MPA), or 200 nM antimycin (AA), and virus titers in tissue culture supernatants were determined by plaque assay at 24 hpi. Results represent the mean ± SEM from three independent experiments. *p*-value < 0.05* or 0.005** compared to DMSO-treated controls. (C) Antimycin A activity against SeV. Vero cells were infected with GFP-SeV at the indicated MOI, treated simultaneously with DMSO or 200 nM antimycin A, and GFP fluorescence was measured at 48 hpi (left graph) and 72 hpi (right graph). There was minimal detectable fluorescence above baseline at 24 hpi (data not shown). Results represent the mean ± SEM from three independent experiments. **p-value < 0.005 compared to DMSO-treated controls. Similar results were obtained with BE(2)-C cells (data not shown). (D) Antimycin A activity against HCV. Huh-7 cells expressing a stable *Renilla* LUC-containing HCV replicon were incubated with decreasing concentrations of antimycin A in the absence of selection, and LUC activity was measured 24 h later. The calculated IC_50_ value for antimycin A in the HCV replicon system is shown on the graph. Results represent the mean ± SEM from three independent experiments.

The initial HTS and validation protocol involved a step that tested extracts in CPE-reduction assays against several viruses, including WEEV, FMV, LACV, and EMCV (Figure 1), which increased the probability of selecting compounds with broad spectrum antiviral activity. The identification of antimycins A1a and A10a as active antiviral components in the S. *kaviengensis* extract confirmed this approach, as dengue virus [37], influenza virus [38], and porcine reproductive and respiratory syndrome virus [36] are all susceptible to antimycin A inhibition. However, these published studies are limited in scope, and therefore we analyzed the breadth of antimycin A antiviral activity against a panel of RNA viruses from the *Togaviridae* (VEEV), *Bunyaviridae* (LACV), *Picornaviridae* (EMCV), *Rhabdoviridae* (vesicular stomatitis virus, VSV), *Paramyxoviridae* (Sendai virus, SeV), and *Flaviviridae* (hepatitis C virus, HCV) families (Figure 7). For VEEV, LACV, EMCV, and VSV we used infectious non-recombinant virus and measured the effect of antimycin A on virus production in BE(2)-C and Vero cells. In initial time course experiments, all four viruses showed peak production by 24 hpi at an MOI = 0.1, although the titer magnitude varied by almost 10,000-fold from 10^6^ pfu/ml for EMCV to 10^10^ pfu/ml for VEEV (Figure 7A). Antimycin A suppressed the production of infectious VEEV, LACV, and EMCV in BE(2)-C cells (Figure 7B, left graph), and both VEEV and LACV in Vero cells (Figure 7B, right graph). The lack of antimycin A activity against VSV in either cell type or EMCV in Vero cells was not due to intrinsic cellular or viral resistance, as mycophenolic acid suppressed virus production for all four viruses in both cell lines. Antimycin A also suppressed recombinant GFP-SeV replication at multiple inocula in both Vero (Figure 7C) and BE(2)-C cells (data not shown), and potently inhibited HCV replicon activity in Huh-7 cells (Figure 7D). Taken together, these results indicated that antimycin A had broad spectrum antiviral activity against a wide range of RNA viruses.

### Antimycin A improves clinical disease severity, reduces mortality, and decreases CNS viral titers in mice infected with WEEV

**Figure 8:**
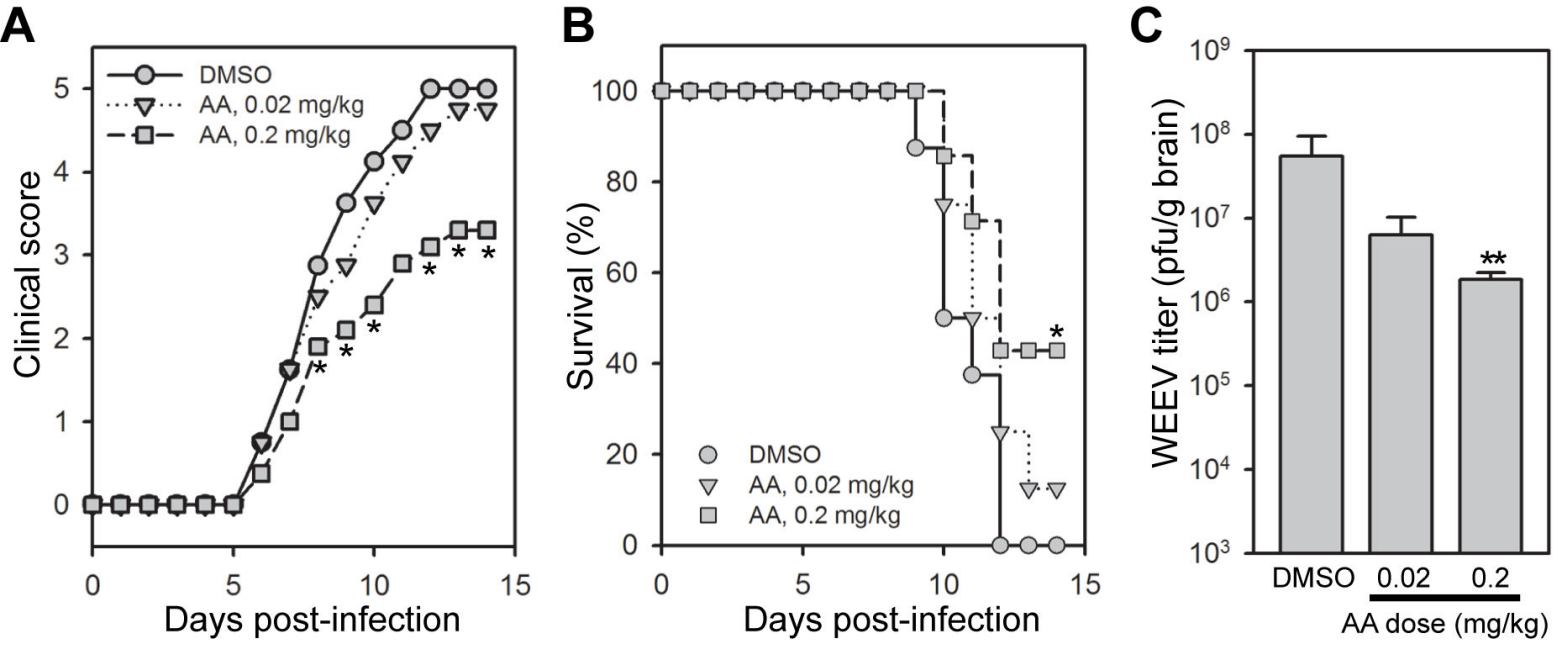
Antimycin A improves clinical disease and survival and reduces CNS titers in mice infected with WEEV. (A and B) Clinical disease severity and survival in WEEV-infected mice. C57BL/6 mice were infected with 10^3^ pfu WEEV, treated twice daily with DMSO or the indicated dose of antimycin A via intraperitoneal injection, and both clinical disease (A) and mortality (B) were monitored for 14 days post-infection. Representative results from one of two independent experiments are shown (N = 7-8 mice per group). *p-value < 0.05 compared to DMSO-treated mice. (C) Virus titers in the CNS of WEEV-infected mice. Mice were infected and treated as described above, and virus titers in brain were determined at 6 days post-infection. N = 4 mice per group. **p-value < 0.01 compared to DMSO control.

Despite the anticipated in vivo toxicity of mETC inhibitors, the high selectivity index of antimycin A in vitro (~48,000, Figure 6B) raised the possibility that we might be able to discern a therapeutic window in vivo. Thus, we examined the antiviral activity of antimycin A in mice infected with WEEV (Figure 8). The recombinant Cba87 strain of WEEV is highly pathogenic in mice [56], and initial dose-titration experiments indicated that a subcutaneous inoculum of 10^3^ pfu routinely produced high mortality in C57BL/6 mice by 14 days after infection with a mean time to death (MTD) of approximately 11 days (data not shown). Antimycin A toxicity is species-dependent and varies widely, from an oral LD_50_ of less than 0.2 mg/kg for fish to greater than 50 mg/kg for mice [57]. The intraperitoneal LD_50_ for mice is 1-2 mg/kg [57,58], and therefore we tested three doses: 1 mg/kg, 0.2 mg/kg, and 0.02 mg/kg delivered via intraperitoneal injection twice daily for 7 days starting on the day of infection. Initial experiments showed that 1 mg/kg antimycin A accelerated WEEV-induced disease with an MTD of approximately 7 days (data not shown). However, when antimycin A doses were reduced by 5- and 50-fold, we were able to discern a modest therapeutic effect. Vehicle-treated control mice showed 100% mortality by 12 days after infection, whereas both clinical disease severity (Figure 8A) and overall survival (Figure 8B) were improved in mice treated with the 0.2 mg/kg antimycin A dose. The lower dose had less clinical effect. We also examined the impact of antimycin A on CNS virus titers in infected mice. Initial experiments showed that CNS titers peaked 5-7 days after infection with WEEV in untreated mice (data not shown). Mice treated with 0.2 mg/kg antimycin A showed a >10-fold reduction in WEEV CNS titers (Figure 6C), while the lower dose resulted in a smaller titer reduction that approached statistical significance (*p*-value = 0.065). These results indicated that antimycin A was effective in improving clinical disease, prolonging survival, and reducing CNS virus titers in mice with WEEV encephalitis.

## Discussion

The immense biosynthetic capability of microorganisms presents an unrivaled opportunity to explore the boundaries of chemical space in the discovery of novel therapeutic agents. In this report, we took advantage of this capability and used marine microbe-derived extracts combined with a phenotypic cell-based bioassay-guided fractionation process to identify potential novel antiviral compounds. We drew six main conclusions from our studies: (i) the marine actinomycetes *S. kaviengensis* produced a readily isolated antiviral compound, antimycin A1a, which displayed potent activity against WEEV RNA replication and virion production; (ii) antiviral activity extended to at least five closely related antimycin A analogues, although a 2-methoxy derivative with no mETC inhibitory activity was completely inactive as an antiviral compound; (iii) disruption of host mETC activity, either with antimycin A or several unrelated mETC complex inhibitors, suppressed WEEV replication; (iv) the molecular mechanism whereby antimycin A disrupted WEEV replication through mETC inhibition involved, in part, suppression of de novo pyrimidine synthesis; (v) antimycin A showed broad in vitro antiviral activity against alphaviruses, bunyaviruses, picornaviruses, paramyxoviruses, and flaviviruses; and (vi) antimycin A was active in vivo in WEEV-infected mice, albeit with a narrow therapeutic window. These results demonstrate that marine *Streptomyces* produce metabolites with potent antiviral activity, and that sequential fractionation of complex microbe-derived mixtures guided by a phenotypic cell-based assay is a viable approach to broad spectrum antiviral drug discovery.

Natural products have been a cornerstone of drug discovery throughout the history of medicine, and have been a particularly rich source of novel therapeutics to treat infectious diseases and cancer [24]. Although natural products have not been used extensively in the search for new antiviral agents, several compounds derived from marine organisms over the past decade have been shown to contain some degree of antiviral activity [59–61]. Medicinal plant extracts have also been used to identify potential novel antiviral compounds, including some targeted against WEEV [62]. Of particular relevance to the results in this report, several *seco*-pregnane steroids produced by the Chinese herbs *Strobilanthes cusia* and *Cynanchum paniculatum* have in vitro and in vivo activity against several alphaviruses [14]. However, in contrast to our results with *S. kaviengensis*-derived antimycin A demonstrating broad spectrum activity, these plant-derived steroids, whose precise target and molecular mechanism of action remain unknown, selectively suppress subgenomic RNA synthesis and show restricted antiviral activity limited to alphavirus-like RNA viruses [14].

We chose an unbiased phenotypic cell-based assay rather than a targeted assay against a specific viral or cellular protein to select and validate extracts and guide fractionation in an effort to broaden the potential targets and mechanisms of action for candidate antivirals. The use of a phenotypic cell-based antiviral assay also increases the probability of selecting compounds having a cellular rather than a viral target, thereby potentially broadening the antiviral spectrum and presenting a higher barrier against the development of viral resistance [63]. Although the phenotypic approach has been successful in the discovery and development of pharmaceuticals [64], there are several potential disadvantages of these assays, including an often lengthy and complex series of studies to determine molecular mechanism of action once a functional chemical entity has been purified and validated. We had the advantage of isolating an antiviral compound from *S. kaviengensis* whose molecular activity and cellular target have already been described. Although the general molecular mechanism for antimycin A activity has been known for decades, the downstream consequences of mETC disruption are numerous and include changes in cellular processes that could have unpredictable effects on virus replication. For example, mETC inhibition with nanomolar concentrations of antimycin A or myxothiazole suppresses cellular autophagy [65], a cellular recycling process associated with the replication of several viruses [66]. Furthermore, mETC inhibition can generate mitochondrial ROS or suppress ATP production, both of which could directly or indirectly inhibit virus replication. We found no evidence for global ROS generation in cells treated with the same concentrations of antimycin A that actively suppressed virus replication, and antioxidants did not reverse antimycin A-mediated antiviral activity. We also found no correlation between global ATP production and the antiviral activity of antimycin A, in contrast to its proposed mechanism for suppression of influenza virus replication [38]. However, we cannot exclude the impact of antimycin A on localized ATP levels at subcellular sites of viral RNA synthesis, which may be a more important determinant of antiviral activity [67].

We were able to rescue antimycin A-mediated antiviral activity with uridine supplementation, suggesting that de novo pyrimidine synthesis, a process tightly linked to mETC activity via DHODH [55], was partially responsible for the antiviral activity of antimycin A against WEEV. This observation is consistent with the identification of specific small molecule DHODH inhibitors as a potent broad spectrum antiviral compounds [52], including activity against WEEV [53]. However, we did not observe antimycin A activity against VSV, even though other groups have shown potent activity of specific DHODH inhibitors against this rhabdovirus in cultured cells [52,53]. Since we did not complete full titration analyses with VSV and antimycin A, we cannot exclude lower potency activity that may have been missed by our experimental approach. In addition, we demonstrate in vivo activity of antimycin A in mice infected with WEEV, whereas DHODH inhibitors are not active in mice [53]. These discrepancies may be due to virus-specific effects or differences in pharmacokinetic parameters between small molecule DHODH inhibitors and antimycin A. Alternatively, antimycin A-mediated suppression of de novo pyrimidine biosynthesis may only partially account for its potent antiviral activity, and modulation of additional cellular pathways downstream of mETC inhibition may also be involved.

The direct clinical utility of unmodified antimycin A as an antiviral agent will likely be limited by toxicity. However, some antimycin A derivatives have favorable pharmacokinetic parameters, including a prolonged terminal half-life in serum [43]. In addition, both our in vivo results in WEEV-infected mice and early toxicology studies [58] suggest some level of antimycin A penetration into the CNS, a crucial requirement for therapies directed against neurotropic pathogens. Detailed structure-activity analyses focused on the antiviral activity of antimycin A have not been conducted, and therefore it would be premature to exclude further development or repurposing of this compound or its derivatives for therapeutic use as broad spectrum antiviral agents. Studies are currently in progress to define the structural requirements for antimycin A activity against WEEV in vitro and in vivo, and to examine potential synergistic activity with established neuroprotective agents or other unrelated compounds active against WEEV [13]. Furthermore, the observation that antimycin A, myxothiazole, rotenone, CCCP, and oligomycin all disrupted WEEV replication suggests that the cellular mETC is a candidate drug target for the development of broadly active antiviral compounds. There is ample evidence supporting mitochondrial targeting in drug discovery and development, and the mETC has been shown to be an eminently druggable target. Atovaquone, an antimalarial drug in clinical use, specifically inhibits the parasite mETC, which along with pyrimidine biosynthesis is the target of several additional antimalarial compounds currently under development [68]. Furthermore, human mETC-targeted drugs represent an important and growing component of potential novel anticancer therapies, as malignant cells are often more sensitive to mitochondrial destabilization [69]. Although the goals of therapy for cancer and infectious diseases can be quite divergent, there is also striking precedence for their inadvertent convergence with respect to drug discovery [70,71].

In summary, we describe a comprehensive and successful antiviral drug discovery program from the initial isolation of a novel marine *Streptomyces* species to the bioassay-directed purification, identification, in vitro characterization, and in vivo validation of a secondary metabolite with potent and broad spectrum antiviral activity. Our studies highlight the tremendous potential of harnessing the chemical diversity inherent in natural products derived from marine microbes as source material for antiviral drug discovery.

## Materials and Methods

### Ethics statement

All animals were housed and used on-site under specific pathogen-free conditions in an approved animal BSL3 facility in strict accordance with guidelines set by the National Institutes of Health and protocols approved by the University of Michigan Committee on the Use and Care of Animal and the Institutional Biosafety Committee (Protocol number 10055-2).

### Marine microbe culture, fermentation, and extract preparation

Marine microbes were collected, isolated, and extracts for HTS were prepared as previously described [30]. We initially completed optimization experiments to maximize antiviral compound production using small scale cultures with six different media (ISP2, X1, A3M, A3M spiked with *Rhodococcus erythropolis* [72], ISP2 without NaCl, and ISP2 agar). ISP2 media resulted in the best production of biologically active extracts with antiviral activity (data not shown), and therefore was chosen for large scale production. An oatmeal plate (6% oat meal, 1.25% agar, NaCl) was streaked from a glycerol stock and incubated 5 days. Seed cultures of 3 ml ISP2 media (1% malt extract, 0.4% yeast extract, 0.4% dextrose, 3% NaCl) were inoculated with a loop full of vegetative cells from an oatmeal plate culture of *S. kaviengensis* and incubated with shaking (200 rpm) at 28°C for 5 days, and subsequently transferred to 100 ml cultures for the same incubation conditions.

For large scale production, 25 ml of the seed cultures were transferred to a 2.8 L Fernbach flask containing 1.5 L of ISP2 and incubated on a rotary shaker (200 rpm) at 28°C for 4 days. This process was performed twice with 16 Fernbach flasks (total culture 24 L each time). After 4 days the cultures were harvested by centrifugation and the resulting cell-free supernatant was subjected to solid phase extraction using 20 g Amberlite XAD-16N resin per L of culture. The resin was separated by filtration and subjected to organic extraction using different organic solvents. The first large scale culture was extracted three times: twice with methanol and once with 1:1 methanol:ethyl acetate mixture to provide a final yield of 8.2 g dried crude extract. The second large scale culture was sequentially extracted with methanol, ethyl acetate, acetone, and hexanes to provide a final yield of 14.0 g dried crude extract.

### *Streptomyces*-derived antiviral compound purification and structure determination

Organic extracts from *S. kaviengensis* were evaporated to dryness, dissolved in 100 ml H_2_O, and loaded onto a C_18_ silica gel column (30 x 2.6 cm, YMC Gel ODS-A, 12 nm, S-150 µm). The C_18_ column was eluted with a stepwise gradient of H_2_O/acetonitrile (100:0 → 0:100) to give eight fractions, designated F1 through F8, and column wash with ethyl acetate. All fractions were concentrated under vacuum and assayed for antiviral activity using the WEEV replicon assay. Fraction F7 (15:85 H_2_O/acetonitrile) showed the highest potency, whereas fractions F5, F6, and F8 had lesser potency. Fraction F7 (143 mg) was subjected to a Sephadex LH-20 column in 1:1 chloroform/methanol to obtain eight fractions, designated F7A through F7H, and a column wash, designated F7I. Two fractions, F7D and F7E, showed the most activity. Fraction F7E (12.4 mg) was separated on a reversed phase HPLC column (Waters XBridge, 250 x 20 mm, 5 mm, 17:83 H_2_0/acetonitrile, DAD at 210, 238 and 254 nm, flow rate 5 ml/min) eluted with 1:9 H_2_0/acetonitrile to give eight fractions, designated F7E1 through F7E8. Fraction F7E2 showed the most potent antiviral activity, and was further separated using a different reversed phase HPLC column (Alltech Econosil C_18_, 5 mm x 250 mm, 22.5 mm, 17:83 H_2_0/acetonitrile, DAD at 210, 238 and 254 nm, flow rate 5 ml/min), to yield six fractions, designated F7E2a through F7E2f. HPLC separations were performed with an Agilent 1100 HPLC system.

Fraction F7E2e (t_R_ = 47.3 min, 2.5 mg, 0.003% of the initial dried crude extract) was a pure compound, determined by LC-MS analysis, and was highly active in the WEEV replicon assay with an IC_50_ of approximately 3 nM (see Table 1). The second extract was fractionated using the same procedure described above to produce approximately 12 mg of the same molecule that was 0.0085% of the initial dried crude extract. This pure compound was identified by NMR as antimycin A1a. In addition, from the second extract another pure compound was isolated, fraction F7E2f (t_R_ = 98.3 min, 8.3 mg, 0.0059% of the initial dried crude extract), which was identified by NMR as antimycin A10a (see Figure 5A) and also showed potent antiviral activity with a WEEV replicon assay IC_50_ of approximately 3 nM. (data not shown). Low resolution LC-MS analyses of HPLC fractions were completed using a Shimadzu 2010 EV APCI spectrometer, and high-resolution APCI-MS spectra were measured using an Agilent Q-TOF HPLC-APCI-MS. NMR spectra were acquired in a Varian INOVA 600 MHz NMR Facility.

### Inhibitors and Antioxidants

Ribavirin, mycophenolic acid, antimycin A, myxothiazole, rotenone, thenoyltrifluoroacetone, oligomycin, CCCP, 2-MPG, and *N*-acetyl cysteine were purchased from Sigma-Aldrich (St. Louis, MO), and 2-methoxyantimycin A3 was purchased from Enzo Life Sciences (Farmingdale, NY).

### Viruses

The Cba-87 strain of WEEV was generated from the cDNA clone pWE2000 as previously described [73]. EMCV and the CM4-146 strain of FMV were purchased from the American Type Culture Collection (Manassas, VA). The human/1960 strain of LACV and the TC-83 vaccine strain of VEEV were obtained from Robert Tesh (University of Texas Medical Branch, Galveston, TX), and the Indiana-1 strain of VSV was obtained from Katherine Spindler (University of Michigan, Ann Arbor, MI). GFP-tagged SeV was obtained from Valery Grdzelishvili (University of North Caroline Charlotte, Charlotte, NC) and has been previously described [74]. All experiments with infectious WEEV were performed under BSL3 conditions in accordance with University of Michigan Institutional Biosafety Committee, Centers for Disease Control, and National Institutes of Health guidelines.

### Cell culture

BE(2)-C, BHK-21, Vero, CHO, SH-SY5Y, and U87 cells were purchased from the American Type Culture Collection. HEK293 cells were obtained from David Markovitz (University of Michigan, Ann Arbor, MI), Huh-7 cells were obtained from Raymond Chung (Massachusetts General Hospital, Boston, MA), BHK-21 cells stably expressing bacteriophage T7 RNA polymerase (BSR-T7 cells) were obtained from Klaus Conzelman (Max von Pettenkofer-Institut, Munich, Germany) and Sonja Gerrard (University of Michigan, Ann Arbor, MI), BHK-21 cells stably expressing an EEEV replicon were obtained from Ilya Frolov (University of Alabama - Birmingham, Birmingham, AL), and Huh-7 cells stably expressing an HCV replicon were obtained from Nobuyuki Kato (Okayama University, Okoyama, Japan). BSR-T7 cells were cultured as previously described [13], and all other cells were cultured in Dulbecco’s Modified Eagle Medium containing 5% bovine growth serum, 1% sodium pyruvate, 0.1 mM non-essential amino acids, 10 U/ml penicillin, and 10 μg/ml streptomycin.

### Virus replication assays

WEEV replicon assays used the plasmid pWR-LUC and BSR-T7 cells and measured firefly LUC accumulation as a surrogate marker for viral RNA replication as previously described [13,18]. The HTS with the microbe-derived extract library was completed in a 384-well plate format as previously described [18]. Infectious titers for all viruses were determined by plaque assay on Vero cell monolayers as previously described [73] with the following modifications. Cells were overlaid with a 1.2% (wt/vol) colloid suspension of AviCell R-581 (FMC Biopolymer, Philadelphia, PA) in complete media rather than agarose, and cells were harvested at 36-40 hpi for WEEV, VEEV, VSV and 48-52 hpi for EMCV and LACV. GFP-SeV replication was determined by GFP accumulation as previously described [74]. Quantitative RT-PCR was done as previously described [18,74,75], and the sequences for the 18S rRNA and WEEV envelope glycoprotein 1 primers have been previously published [75]. The sequences for the forward and reverse WEEV nsP1 qRT-PCR primers were 5’-GCAGTCCATGCACCGACA-3’ and 5’-GGCTGGTACATACGTACA-3’, respectively. Northern blotting with ^32^P-labeled riboprobes was done as previously described [73]. HCV replicon assays used *Renilla* LUC accumulation and were done in stable expressing Huh-7 cells as previously described [76].

### Cell viability, ATP, and ROS assays

Cell viability after viral infection or drug treatment was determined by MTT assay as previously described [73]. Total cellular ATP levels were determined using the ATPlite assay (PerkinElmer, Waltham, MA) according to the manufacturer’s instructions. Cellular ROS levels were determined with the general oxidative stress indicator 5(6)-chloromethyl-2’,7’-dichlorodihydrofluoroscein diacetate (CM-H_2_DCFDA) according to the manufacturer’s instructions (Life Technologies, Grand Island, NY).

### Microarray and pathway analysis

Transcriptome analyses were done on three independent sets of cultures for each comparison using Affymetrix Human U133 Plus 2.0 microarray chips as previously described [74]. Complete original data files have been deposited in the Gene Expression Omnibus database (www.ncbi.nlm.nih.gov/geo) under accession number GSE44541. The Genomatix ChipInspector software package (Genomatix Software Inc., Ann Arbor, MI) was used for primary microarray data analysis. The following parameters were chosen to identify sets of differentially regulated transcripts: (i) false-discovery rate of 1%; (ii) three probe minimum coverage; and (iii) expression level log_2_ change ≥ 1 (2-fold) compared to control. The list of genes preferentially up- or down-regulated in BE(2)-C cells treated with F7E2e or commercial antimycin A were analyzed using Ingenuity Pathway Analysis software (Ingenuity Systems, Redwood, CA). Significance was measured by determining the ratio of the number of genes from the data set that map to a particular canonical pathway to the total number of genes for that pathway, and calculating a subsequent *p*-value using a Fischer’s exact test. The association with a particular canonical pathway was considered significant if the *p*-value was < 0.05.

### Animal infection and treatment experiments

Female C57BL/6 mice (5-6 weeks of age) were purchased from the Jackson Laboratory (Bar Harbor, ME). Mice were housed on a 10/14 h light/dark cycle in ventilated cages containing no more than five animals per cage, and food and water were available ad libitum. For WEEV infection, mice were inoculated subcutaneously with 10^3^ pfu WEEV suspended in 100 μl PBS. Antimycin A was solubilized in DMSO as a stock solution at 100 mM and diluted in PBS to generate working solutions for intraperitoneal injections into infected mice on a twice-daily dosing schedule for 7 days following virus inoculation. Mice were followed for an additional 7 days after treatment was completed, and all remaining animals were euthanized at 14 days after infection. Daily weights were obtained on all mice, and clinical scoring was done using a 1 to 5 scale as previously described [13]. Virus titers in brain tissue were determined by plaque assay as described above.

### Statistical analysis

We used a two-tailed Student’s *t*-test assuming equal variances for routine comparative analyses, and we performed statistical analyses on log_10_-transformed virus titer data. Microarray and pathway statistical analyses are described above. Differences in survival among cohorts of WEEV-infected mice were measured using a log-rank (Mantel-Cox) test. In all cases, differences at a *p*-value < 0.05 were considered significant.

## Supporting Information

Figure S1 **05-1015-2N strain identification and phylogenetic analysis.**
(PDF)

Figure S2 **Validation of WEEV replicon-guided fractionation as a surrogate for antiviral activity.**
(PDF)

Figure S3 **Effect of mycophenolic acid and purified antiviral compound F7E2e from *S. kaviengensis* on viability and proliferation of various cultured cell lines.**
(PDF)

Figure S4 **Correlation between transcriptional responses in BE(2)-C cells treated with *S. kaviengensis*–derived F7E2e or commercial antimycin A.**
(PDF)

Table S1 **NMR data for structure elucidation of antimycin A derivatives dissolved in CDCl_3_.**
(DOCX)

Table S2 **BE(2)-C transcriptional response to *S. kaviengensis*-derived compound F7E2e.**
(XLSX)

Table S3 **BE(2)-C transcriptional response to commercial antimycin A.**
(XLSX)

Table S4 **Comparison of BE(2)-C transcriptional responses to *S. kaviengensis*-derived compound F7E2e or commercial antimycin A.**
(XLSX)

Table S5 **Ingenuity Pathway Analysis (IPA) of BE(2)-C transcriptional responses to *S. kaviengensis*-derived compound F7E2e or commercial antimycin A.**
(XLSX)

Table S6 **Ingenuity Pathway Analysis (IPA) mitochondrial dysfunction pathway genes upregulated in BE(2)-C cells stimulated with *S. kaviengensis*-derived compound F7E2e or commercial antimycin A.**
(XLSX)

Table S7 **Synergy-antagonism assay results of antimycin A and other mitochondrial electron transport chain inhibitors.**
(DOCX)
