# Supplementary material for: Discovery of Potent Broad Spectrum Antivirals Derived from Marine Actinobacteria
Source: PLoS One. 2013 Dec 5;8(12):e82318. doi: 10.1371/journal.pone.0082318 (PMC3857800; doi:10.1371/journal.pone.0082318)
Supplement: Figure S1 — 05-1015-2N strain identification and phylogenetic analysis. (PDF) [file pone.0082318.s001.pdf]

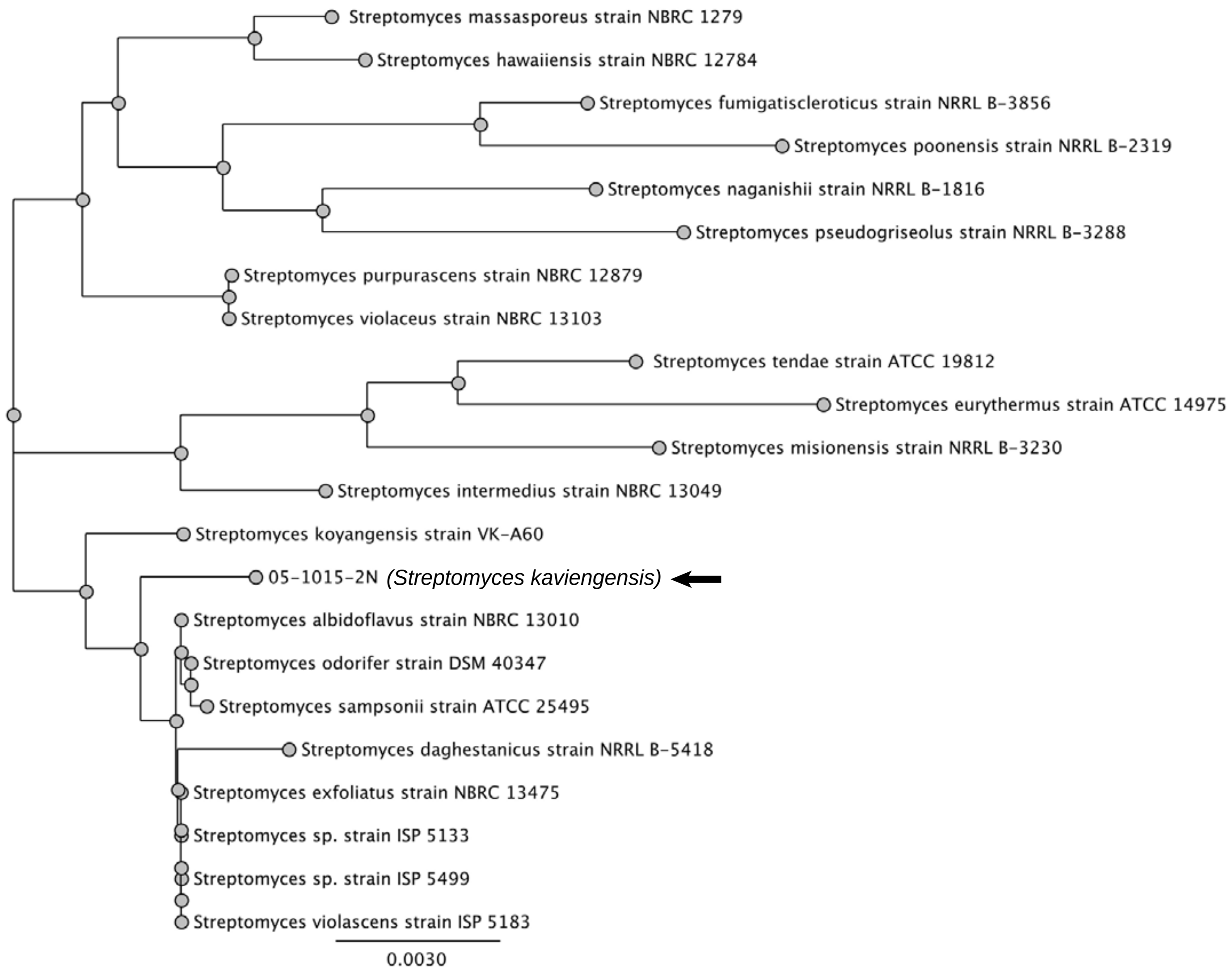

Figure S1. 05-1015-2N strain identification and phylogenetic analysis. Genomic DNA from strain 05-1015-2N encoding the 16S rRNA was amplified by PCR using the universal primers FC27 (5′-AGAGTTTGATCCTGGCTCAG-3′) and RC1492 (5′-TACGGCTACCTTGTTACGACTT-3′), cloned into pGEM (Promega, Madison, WI), and sequenced with T7 and SP6 primers. Phylogenetic analysis based on the 16S rRNA sequence was conducted with GENEIOUS pro version 4.8.4 available from http://www.geneious.com. The bootstrap consensus tree shown was inferred from 1000 replicates using the Neighbor-Joining method, and was taken to represent the evolutionary history of the taxa analyzed. The location of 05-1015-2N (named *Streptomyces kaviengensis*) is indicated by the arrow.
