# Supplementary material for: Discovery of Potent Broad Spectrum Antivirals Derived from Marine Actinobacteria
Source: PLoS One. 2013 Dec 5;8(12):e82318. doi: 10.1371/journal.pone.0082318 (PMC3857800; doi:10.1371/journal.pone.0082318)
Supplement: Figure S2 — Validation of WEEV replicon-guided fractionation as a surrogate for antiviral activity. (PDF) [file pone.0082318.s002.pdf]

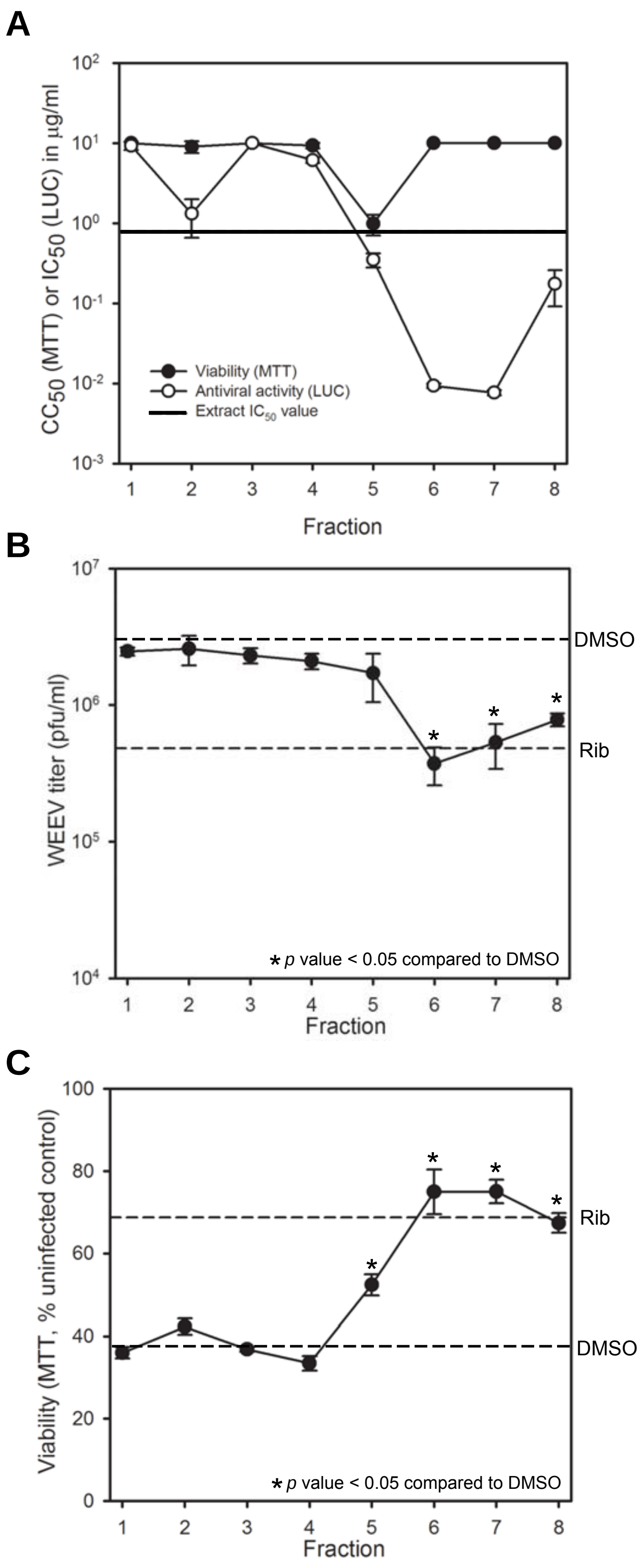

Figure S2. Validation of WEEV replicon-guided fractionation as a surrogate for antiviral activity. (A) Replicon suppression activity of *S. kaviengensis* fractions. Individuals fractions from step 1 in Figure 2A (C18 flash chromatography) were tested for antiviral activity by the WEEV replicon assay and viability by MTT assay in BSR-T7 cells. Full weight-based dose-titration curves were generated for each fraction, and results are presented as IC50 (replicon) or CC50 (viability) values and represent mean ± SEM from three independent experiments. The solid reference line represents the replicon suppression IC50 value for the unfractionated extract from *S. kaviengensis.* (B and C) Antiviral activity of extract fractions measured by suppression of WEEV production (B) or rescue of WEEV-induced CPE (C). BE(2)-C cells were infected with WEEV at an MOI = 0.1, treated with 1 µg/ml of the indicated fraction from step 1 in Figure 2A, and both infectious virion titers in tissue culture supernatants by plaque assay (B) and viability by MTT assay (C) were measured at 24 hpi. The dashed lines represent results from infected cells treated with the negative or positive controls, DMSO or 50 µM ribavirin (Rib), respectively.
