# Supplementary material for: Discovery of Potent Broad Spectrum Antivirals Derived from Marine Actinobacteria
Source: PLoS One. 2013 Dec 5;8(12):e82318. doi: 10.1371/journal.pone.0082318 (PMC3857800; doi:10.1371/journal.pone.0082318)
Supplement: Figure S3 — Effect of mycophenolic acid and purified antiviral compound F7E2e from S. kaviengensis on viability and proliferation of various cultured cell lines. (PDF) [file pone.0082318.s003.pdf]

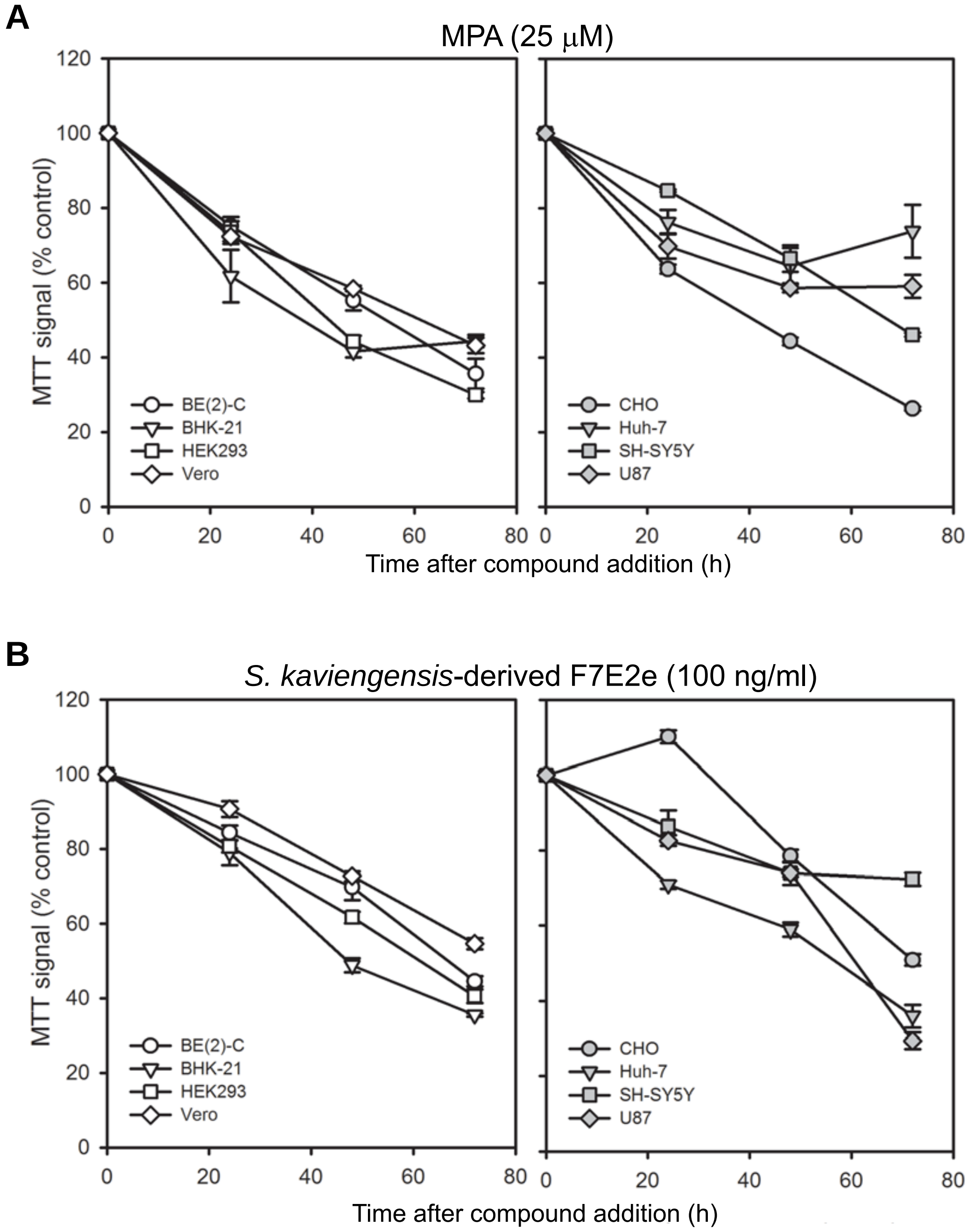


**Figure S3.** Effect of mycophenolic acid and purified antiviral compound F7E2e from *S. kaviengensis* on viability and proliferation of various cultured cell lines. The indicated cell lines were treated with either 25 μM mycophenolic acid (MPA) (A) or 100 ng/ml (~200 nM) *S. kaviengensis* compound F7E2e (B), and cell viability was measured by MTT assay at 24, 48, and 72 h after compound addition. Results represent the mean ± SEM from three independent experiments.
