# Supplementary material for: Discovery of Potent Broad Spectrum Antivirals Derived from Marine Actinobacteria
Source: PLoS One. 2013 Dec 5;8(12):e82318. doi: 10.1371/journal.pone.0082318 (PMC3857800; doi:10.1371/journal.pone.0082318)
Supplement: Figure S4 — Correlation between transcriptional responses in BE(2)-C cells treated with S. kaviengensis–derived F7E2e or commercial antimycin A. (PDF) [file pone.0082318.s004.pdf]

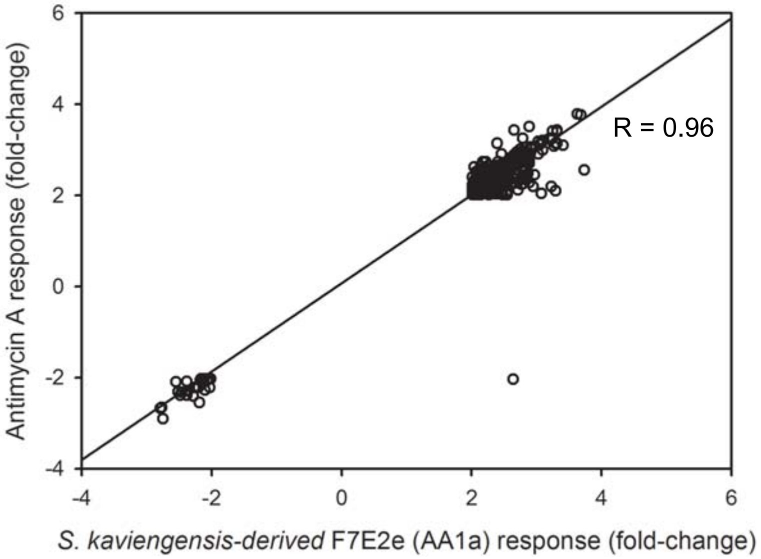

Figure S4. Correlation between transcriptional responses in BE(2)-C cells treated with *S. kaviengensis*–derived F7E2e or commercial antimycin A. Human BE(2)-C neuronal cells were treated with 100 ng/ml (~200 nM) F7E2e or commercial antimycin A, total RNA was harvested 24 h later, and transcriptional responses were analyzed using Affymetrix Human U133 Plus 2.0 arrays and Genomatix software. Complete lists of up- and down-regulated genes are given in Tables S2 and S3, and comparisons of co-regulated genes are provided in Table S4. The 757 genes that were co-regulated after treatment with F7E2e or commercial antimycin A and demonstrated a ≥2-fold change are shown.
