# Supplementary material for: Discovery of Potent Broad Spectrum Antivirals Derived from Marine Actinobacteria
Source: PLoS One. 2013 Dec 5;8(12):e82318. doi: 10.1371/journal.pone.0082318 (PMC3857800; doi:10.1371/journal.pone.0082318)
Supplement: Table S1 — NMR data for structure elucidation of antimycin A derivatives dissolved in CDCl3. (DOCX) [file pone.0082318.s005.docx]

**Supplemental Table S1. NMR data for antimycin A derivative structure elucidation.**

| Atom | Antimycin A1a^1^ | | Antimycin A2a | | Antimycin A3a | | Antimycin A4a | | Antimycin A10a^2^ | |
| --- | --- | --- | --- | --- | --- | --- | --- | --- | --- | --- |
|  | δ_13C_ | δ_1H_ | δ_13C_ | δ_1H_ | δ_13C_ | δ_1H_ | δ_13C_ | δ_1H_ | δ_13C_ | δ_1H_ |
| 2 | 170.2 | -- | 170.2 | -- | 170.3 | -- | 170.1 | -- | 170.2 |  |
| 3 | 53.8 | 5.32 | 53.8 | 5.30 | 53.8 | 5.30 | 53.7 | 5.29 | 53.7 | 5.29 |
| 4 | 71.1 | 5.75 | 71.0 | 5.73 | 71.0 | 5.74 | 70.9 | 5.74 | 70.9 | 5.74 |
| 4-Me | 15.1 | 1.32 | 15.1 | 1.31 | 15.1 | 1.31 | 14.9 | 1.31 | 14.9 | 1.30 |
| 6 | 173.1 | -- | 173.0 | -- | 173.1 | -- | 172.9 | -- | 173.0 | -- |
| 7 | 50.4 | 2.56 | 50.3 | 2.53 | 50.3 | 2.52 | 50.1 | 2.52 | 50.1 | 2.53 |
| 8 | 75.3 | 5.12 | 75.2 | 5.07 | 75.0 | 5.10 | 75.4 | 5.10 | 75.3 | 5.11 |
| 9 | 74.9 | 4.98 | 75.4 | 4.99 | 74.9 | 4.98 | 74.8 | 5.00 | 74.8 | 4.99 |
| 9-Me | 18.0 | 1.29 | 18.0 | 1.28 | 18.0 | 1.28 | 17.8 | 1.28 | 17.8 | 1.30 |
| 1’-**C**ONH | 169.5 | -- | 169.5 | -- | 169.6 | -- | 169.4 | -- | 169.4 | -- |
| 1’-CON**H** | -- | 7.09 | -- | 7.10 | -- | 7.13 | -- | 7.23 | -- | 7.07 |
| 1’ | 112.7 | -- | 112.7 | -- | 112.9 |  | 112.9 |  | 112.7 | -- |
| 2’ | 120.2 | 7.25 | 120.4 | 7.25 | 120.3 | 7.25 | 120.3 | 7.25 | 120.0 | 7.24 |
| 3’ | 119.1 | 6.92 | 119.1 | 6.91 | 119.3 | 6.91 | 119.3 | 6.91 | 118.9 | 6.90 |
| 4’ | 124.9 | 8.55 | 125.0 | 8.53 | 124.9 | 8.53 | 124.9 | 8.54 | 124.6 | 8.54 |
| 5’ | 127.6 | -- | 127.6 | -- | 127.6 | -- | 127.6 | -- | 127.6 | -- |
| 6’ | 150.8 | -- | 150.8 | -- | 150.9 | -- | 150.9 | -- | 150.8 | -- |
| 6-OH | -- | 12.62 | -- | 12.60 | -- | 12.57 | -- | 12.57 | -- | 12.62 |
| 5’N**H**CO(H) | -- | 7.99 | -- | 7.97 | -- | 7.99 | -- | 7.92 | -- | 7.93 |
| 5’NH**C**O(**H**) | 159.2 | 8.50 | 159.1 | 8.50 | 159.2 | 8.50 | 158.9 | 8.50 | 159.1 | 8.50 |
| α | 28.4 | 1.56 | 28.3 | 1.60 | 28.3 | 1.60 | 28.1 | 1.70 | 28.7 | 1.70 |
| β | 22.6 | 1.27 | 22.4 | 1.26 | 22.7 | 1.28 | 22.4 | 1.18 | 24.4 | 1.25 |
| γ | 27.1 | 1.27 | 27.1 | 1.26 | 29.2 | 1.28 | 29.2 | 1.25 | 36.1 | 1.25 |
| δ | 31.5 | 1.27 | 31.5 | 1.26 | 13.9 | 0.85 | 13.7 | 0.85 | 34.1 | 1.25 |
| ε | 28.9 | 1.27 | 29.0 | 1.26 | -- | -- | -- | -- | 29.5 | 1.25 |
| ζ | 14.2 | 0.85 | 14.0 | 0.85 | -- | -- | -- | -- | 11.4 | 0.77 |
| η | -- | -- | -- | -- | -- | -- | -- | -- | 19.0 | 0.81 |
| 1’’ | 175.3` | -- | 171.5 | -- | 175.5 | -- | 171.5 | -- | 175.3 | -- |
| 2’’ | 41.4 | 2.42 | 36.1 | 2.46 | 41.4 | 2.41 | 36.1 | 2.35 | 41.4 | 2.42 |
| 3’’ | 26.1 | 1.48  1.73 | 26.3 | 1.49  1.75 | 26.6 | 1.48  1.73 | 18.4 | 1.73 | 26.5 | 1.48  1.73 |
| 4’’ | 11.5 | 0.84 | 11.7 | 0.86 | 11.9 | 0.94 | 13.7 | 0.94 | 11.7 | 0.94 |
| 5’’ | 17.0 | 1.19 | 17.1 | 1.21 | 17.1 | 1.18 | -- | -- | 16.7 | 1.10 |

^1^ References for antimycin A1a NMR coordinates: Barrow, C. J.; Oleynek, J. J.; Marinelli, V.; Sun, H. H.; Kaplita, P.; Sedlock, D. M.; Gillum, A. M.; Chadwick, C. C.; Cooper, R. *J. Antibiot.* **1997**, *50*, 729–733; Liu, W.-C.; Strong, F. M. *J. Am. Chem. Soc.* **1959**, *81*, 4387–4390.

^2^ Reference for antimycin A10a NMR coordinates: Hosotani, N.; Kumagai, K.; Nakagawa, H.; Shimatani, T.; Saji, I. *J. Antibiot.* **2005**, *58*, 460–467.
