# Supplementary material for: Discovery of Potent Broad Spectrum Antivirals Derived from Marine Actinobacteria
Source: PLoS One. 2013 Dec 5;8(12):e82318. doi: 10.1371/journal.pone.0082318 (PMC3857800; doi:10.1371/journal.pone.0082318)
Supplement: Table S7 — Synergy-antagonism assay results of antimycin A and other mitochondrial electron transport chain inhibitors. (DOCX) [file pone.0082318.s011.docx]

**Supplemental Table S7. Synergy-antagonism assay results of antimycin A and mitochondrial electron transport and respiratory inhibitors.**

|  |  | **Chou-Talaley Parameter^3^** | | | **Combination index (CI)^4^** | | |
| --- | --- | --- | --- | --- | --- | --- | --- |
| **Compound/Combination^1^** | **Molar ratio^2^** | **Potency (*D_m_*)** | **Shape (*m*)** | **Conformity (*r*)** | **IC_50_** | **IC_75_** | **IC_90_** |
| Antimycin A | NA | 3.3 ± 0.4 nM | -3.60 ± 0.37 | -0.96 ± 0.01 | NA | NA | NA |
| Rotenone | NA | 10.1 ± 1.4 nM | -1.32 ± 0.33 | -0.97 ± 0.02 | NA | NA | NA |
| Myxothiazole | NA | 19.6 ± 2.9 nM | -4.10 ± 0.61 | -0.98 ± 0.01 | NA | NA | NA |
| Oligomycin | NA | 7.4 ± 0.8 nM | -3.08 ± 0.73 | -0.97 ± 0.01 | NA | NA | NA |
| CCCP | NA | 2.3 ± 0.2 μM | -2.30 ± 0.14 | -0.99 ± 0.01 | NA | NA | NA |
| Antimycin A + Rotenone | 1:5 | NA | -1.26 ± 0.08 | -0.99 ± 0.01 | 1.24 ± 0.14 | 1.32 ± 0.33 | 1.36 ± 0.41 |
| Antimycin A + Myxothiazole | 1:5 | NA | -4.23 ± 0.77 | -0.98 ± 0.01 | 1.53 ± 0.16 | 1.54 ± 0.19 | 1.58 ± 0.24 |
| Antimycin A + Oligomycin | 1:1 | NA | -3.08 ± 0.65 | -0.99 ± 0.01 | 1.91 ± 0.29 | 1.83 ± 0.30 | 1.81 ± 0.39 |
| Antimycin A + CCCP | 1:800 | NA | -2.26 ± 0.14 | -0.98 ± 0.01 | 691 ± 65 | 690 ± 71 | 698 ± 96 |

^1^ BSR-T7 cells transfected with pWR-LUC were treated with the indicated compound at a range of concentrations around their individual IC_50_ concentrations, or in a fixed molar ratio around the antimycin A IC_50_ concentration.

^2^ The fixed molar ratios were determined based on IC_50_ concentrations to obtain equipotency concentrations at the IC_50_ level.

^3^ We used the CompuSyn program (available at www.combosyn.com) to calculate Chou-Talaley parameters and combination index (CI) values. Results represent the mean ± SEM from five independent experiments.

^4^ The combination index (CI) is a quantitative measure of synergistic, additive, or antagonistic effects between compounds (Chou, T-C., *Pharmacological Reviews*, 58:621-681, 2006). This parameter indicates synergism with values < 1, nearly additive effects with values near 1, and antagonism with values >1. The range of values is 0 to 1 for synergy, and 1 to ∞ for antagonism, where values >10 indicate very strong antagonism.
